# Psychological distress and lack of PINK1 promote bioenergetics alterations in peripheral blood mononuclear cells

**DOI:** 10.1038/s41598-020-66745-9

**Published:** 2020-06-17

**Authors:** Mariana Grigoruţă, Ruben K. Dagda, Ángel G. Díaz-Sánchez, Alejandro Martínez-Martínez

**Affiliations:** 1 Departamento de Ciencias Químico Biológicas, Universidad Autónoma de Ciudad Juárez, Anillo envolvente Pronaf y Estocolmo s/n, 32310 Ciudad Juárez, Chihuahua Mexico; 2 Department of Pharmacology, University of Nevada, Reno School of Medicine, Reno, NV 89557 United States

**Keywords:** Cell biology, Immunology, Neuroscience, Psychology

## Abstract

Psychological distress induces oxidative stress and alters mitochondrial metabolism in the nervous and immune systems. Psychological distress promotes alterations in brain metabolism and neurochemistry in wild-type (WT) rats in a similar manner as in Parkinsonian rats lacking endogenous PTEN-induced kinase 1 (PINK1), a serine/threonine kinase mutated in a recessive forms of Parkinson’s disease. PINK1 has been extensively studied in the brain, but its physiological role in peripheral tissues and the extent to which it intersects with the neuroimmune axis is not clear. We surmised that PINK1 modulates the bioenergetics of peripheral blood mononuclear cells (PBMCs) under basal conditions or in situations that promote oxidative stress as psychological distress. By using an XF metabolic bioanalyzer, PINK1-KO-PBMCs showed significantly increased oxidative phosphorylation and basal glycolysis compared to WT cells and correlated with motor dysfunction. In addition, psychological distress enhanced the glycolytic capacity in PINK1-KO-PBMCs but not in WT-PBMCs. The level of antioxidant markers and brain-derived neurotrophic factor were altered in PINK1-KO-PBMCs and by psychological distress. In summary, our data suggest that PINK1 is critical for modulating the bioenergetics and antioxidant responses in PBMCs whereas lack of PINK1 upregulates compensatory glycolysis in response to oxidative stress induced by psychological distress.

## Introduction

Chronic exposure to psychological distress alters the feedback inhibition pathway of the hypothalamic-pituitary-adrenal (HPA) axis in tissues that are exposed to high concentrations of corticosteroids^1^. In recent years, our understanding on the physiological contributions of mitochondria in being able to sense alterations in the cell environment, integrate multiple signaling pathways and adapt to oxidative stress in response to the psychological state of animals under homeostatic conditions and under psychological distress is emerging^2^. In the brain, psychologically distressed animals showed diminished mitochondrial content in the midbrain, diminished oxidative phosphorylation (OXPHOS) and glycolysis^3^ in the prefrontal cortex, increased oxidative stress^4,5^, and elevated neuroinflammation^6^. In peripheral tissues, we have observed that psychological distress induces significant oxidative damage in peripheral blood mononuclear cells (PBMCs)^7,8^. The long-term pathological effects of psychological distress have been associated with mental and neurodegenerative disorders including major clinical depression, schizophrenia, Parkinson’s disease (PD), Alzheimer’s disease or dementia^1,9^.

Mitochondrial dysfunction and inflammation play central roles in the pathogenesis of chronic diseases like diabetes mellitus^10^, cardiovascular diseases^11^ or neurodegenerative disorders^12^. A balance between mitochondrial biogenesis and mitophagy is crucial in maintaining a proper cell energy metabolism as it maintains a minimum level of functional and high-quality mitochondria required for neuronal survival^13^. On the other hand, detrimental events that changes this balance alter mitochondrial dynamics, mitochondrial function, and maintenance^3,13,14^.

Phosphatase and tensin homolog-induced kinase 1 (PINK1) is a large atypical serine/threonine (ser/thr) kinase localized to mitochondrial and cytosolic compartments in neurons. PINK1 governs mitochondrial quality control, dendrite outgrowth and neuronal survival^15–17^. PINK1, along with E3 ubiquitin-protein ligase Parkin, have vital roles in stimulating mitophagy and mitochondrial Ca^2+^ influx modulation; the disruption of this pathway leads to mitochondria dysfunction and neurodegeneration, cytopathological events associated with early-onset recessive PD^14,18^. However, a recent *in vivo* study showed that basal mitophagy is independent of PINK1 in different tissues that require a high energy demand^19^. In addition, mitophagy is regulated via other pathways including the serine/threonine kinase AKT pathway^20^. As in PD patients, PINK1 knockouts (PINK1-KO) rats faithfully manifest progressive motor and non-motor symptoms^21,22^, significant loss of dopaminergic neurons in the *substantia nigra* (SN)^22,23^, mitochondrial deficiency^24–27^ and alterations of antioxidant proteins^25^ in the brain starting at two months of age^3^. In contrast to other cell types, neurons are highly vulnerable to neurodegeneration in response to PINK1 deficiency. In PD, the selective vulnerability of midbrain dopamine neurons may be attributed to intrinsic properties of this select neuronal subpopulation (e.g. presence of L-type calcium channels) and their reduced ability of mitochondria to uptake excess calcium due to their low level of mitochondrial content^28^.

Mitochondrial dysfunction, as a consequence of loss of PINK1 function, can be caused by respiratory chain defects as observed in PINK1-KO mouse embryonic fibroblasts^29^. In immune system cells, PINK1 regulates the innate immune response during viral infections^30^, participates in the mitochondrial antigen presentation pathway, and a depletion of PINK1 can elicit inflammatory autoimmune responses^31^. In addition, PINK1 regulates the innate immune response of glial cells, inhibits apoptosis pathways during neuroinflammation^32^, and controls the generation of reactive oxygen species (ROS) mediated via the MAPK pathways in hepatic cells^33^. Moreover, PINK1-deficient myocytes exhibit high mitochondrial membrane potential (MMP) relative to wild type (WT) cells. Like PINK1-deficient neurons, PINK1-deficient myocytes have high glycolysis rates and show impaired mitochondrial respiration^34^.

The brain-derived neurotrophic factor (BDNF) is a neuropeptide implicated in neuronal differentiation, development, protection and maintenance^35^. A low level of BDNF in the serum and brain is correlated with the progression of PD, presumably due to the ability of BDNF in maintaining neuronal survival and the complexity of dendritic trees in dopaminergic neurons from the SN^36–39^. Consistent with this concept, a low level of intracellular BDNF in the SN was observed in postmortem brain tissue from PD patients^36,39^. Moreover, young Parkinsonian (PINK1-KO) rats, which show modest motor dysfunction but significant neurodegeneration of SN neurons compared to WT rats, demonstrated a significant diminished expression of intracellular BDNF in the midbrain^3^. In addition, a decrease in the level of plasma BDNF was associated with impaired motor coordination^40^ and, a low serum level of BDNF is associated with major clinical depression^41^ in PD or with the onset of psychiatric disorders like schizophrenia^42^ or bipolar disorder^43^. Because of its high capacity to traverse the blood brain barrier, BDNF is considered a biomarker that can inform on the metabolic state of the neurons^44^, especially given that immune system cells show high levels of BDNF expression^45^.

Impaired functions of distinct subpopulations of immune system cells may contribute to the etiology of PD. Indeed, the chronic activation of immune cells in the periphery and nervous central system is linked with the onset and progression of PD^46^. Moreover, the exposure to chronic psychological distress induces the release of hormones and neurotransmitters^47^, which leads to changes in the biochemical profile in PBMCs, presumably as a compensatory response to adapt to oxidative stress^7,8^, and induces oxidative stress and neuroinflammation in the area postrema and other brain regions^3,5,6^. Unlike other cell types, PBMCs require a high energy demand to modulate a myriad of metabolically demanding processes including regulation of the HPA axis via cytokines and tumor necrosis factor alpha (TNFα)^48^. In eukaryotic cells, a well equilibrated utilization of “fuels” (e.g. amino acids, lipids or glucose) is required to enhance OXPHOS and glycolysis, and thereby maintain homeostasis^49^. To generate ATP in their resting state, immune system cells predominantly rely on OXPHOS but when they are activated, the PBMCs shift from OXPHOS to aerobic glycolysis to proliferate through a phenomenon termed the “Warburg effect” during oxidative stress^50–52^. Aerobic glycolysis can provide the necessary ATP to maintain the MMP and prevents apoptosis. Indeed, leukocytes can bioenergetically switch from OXPHOS to glycolysis by overstimulating the pentose phosphate pathway, enhancing the glucose uptake and by diminishing the activity of OXPHOS, which leads to an increase in the level of reactive oxygen species (ROS) that is subsequently released to the extracellular space as a mechanism to promote bactericidal activity^53^. Even though it is not an energetically favorable process relative to OXPHOS - as two molecules of ATP are generated from one molecule of glucose- aerobic glycolysis is nevertheless preferred by proliferative cells (e.g. cancer cells) due to the fast-enzymatic capacity/activity of glycolytic enzymes to generate ATP. Indeed, a shift from OXPHOS to glycolysis enables immune system cells to adapt and thrive in a hypoxic environment. Moreover, an increase in glucose uptake can enhance the production of metabolites like NADPH, implicated in immune cell activation and growth^54^.

By using an XF metabolic bioanalyzer, several studies have developed methodologies to measure the bioenergetics of PBMCs as a proxy and a prognostic tool for assessing disease progression and overall health, including in humans afflicted with autism spectrums disorders and myalgic encephalomyelitis/ chronic fatigue syndrome (ME/CFS)^55–57^.

In the present study, we show that depletion of PINK1 robustly elevates mitochondrial respiration and glycolysis with a concomitant decrease in BDNF expression in PBMCs, which phenocopies the neurochemical alterations previously observed in PINK1-KO brains^3^. While exposure to psychological distress did not have an additive effect on their OXPHOS status, PBMCs derived from PINK-KO rats (PINK1-KO-PBMCs) underwent significant metabolic switching from OXPHOS to glycolysis in response to oxidative stress induced by psychological distress. The elevated glycolysis observed in PINK1-KO-PBMCs from stressed rats correlated with an increase in the level of the antioxidant proteins, superoxide dismutase (SOD) 2 and DJ1, presumably to maintain the same level of ATP in response to mitochondrial dysfunction caused by psychological distress-mediated oxidative stress. In addition, the bioenergetic alterations in PBMCs coincided with the onset of motor symptoms in the same cohort of PINK1-KO and WT rats that were untreated or exposed to psychological distress. Our study suggests that PINK1 is indispensable for maintaining proper mitochondrial metabolism in PBMCs and profiling the bioenergetics in PBMCs may be used as a proxy to inform on: 1) the progression of PD pathology and, 2) oxidative stress induced by psychological distress and by PINK1 deficiency.

## Results

### Antioxidant parameters

**Figure 1:**
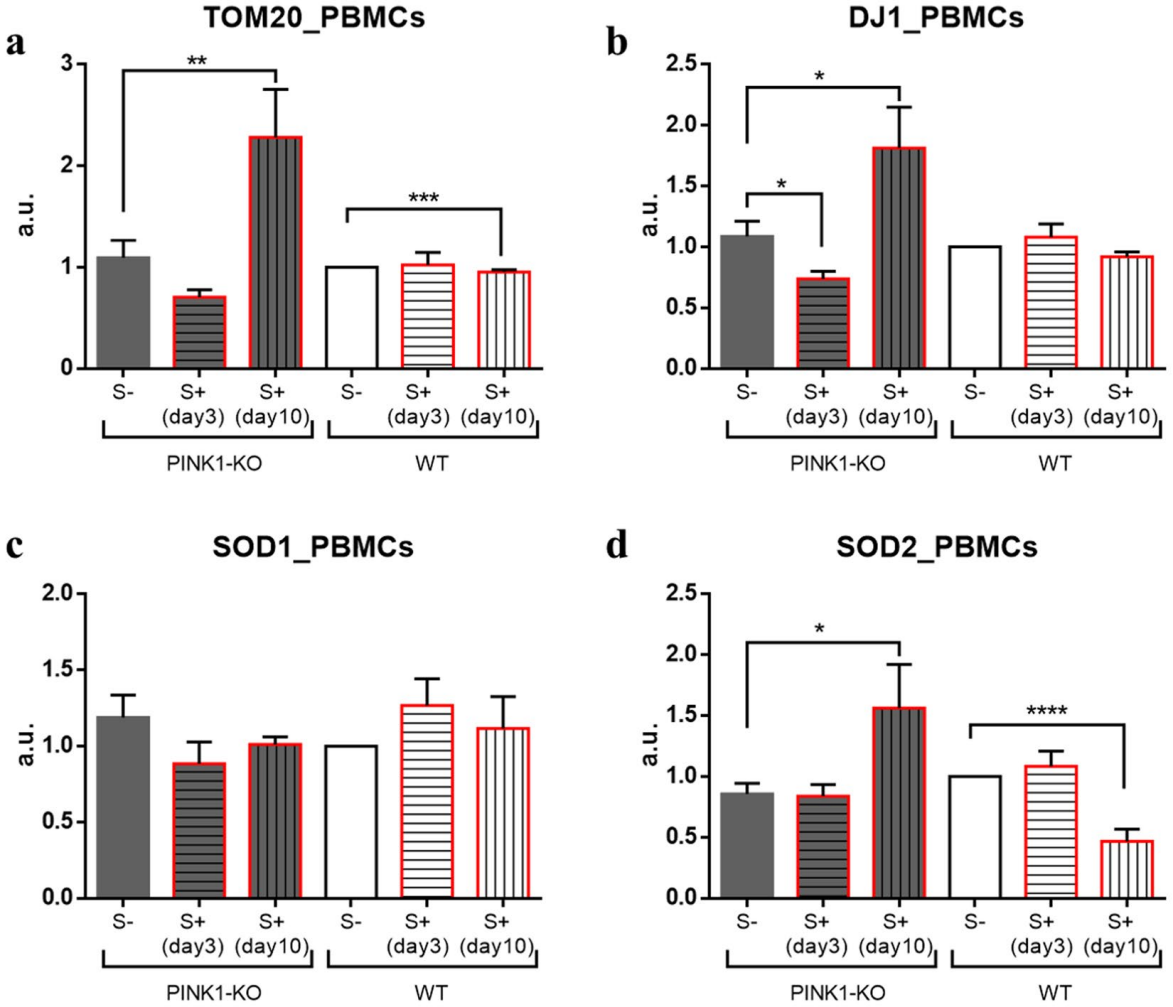
The level of mitochondrial antioxidants and mitochondrial content are elevated in response to psychological distress in PINK1-KO-PBMCs. (**a**) mitochondrial content (TOM20 level), (**b**) DJ-1 level, (**c**) SOD1 level, (**d**) SOD2 level in PBMCs derived from PINK1-KO and WT rats. Raw WB images were added as Supplementary Fig. S1 in the Supplementary Data file. Densitometric analyses of the immunoreactivity for each protein marker were normalized relative to WT S- group. For control groups (S-) n = 13, 4 females and 9 males per phenotype, for stressed groups (S+) sacrificed on day 3 the n = 9, 4 females and 5 males per phenotype, and in day 10 the n = 4 WT males and 5 PINK1-KO males. Data are expressed as mean ± standard error of the mean (SEM); *p ≤ 0.05, **p ≤ 0.01, ***p ≤ 0.001, ****p ≤ 0.0001, two-tailed Student’s t-test.

We have previously observed that psychological distress can induce profound alterations in the level of mitochondria, antioxidant response systems and neurotrophic factors which coincided with bioenergetic alterations, presumably due to the high level of oxidative stress induced by psychological distress^3,6–8^. Likewise, to determine the impact of oxidative stress induced by loss of PINK1 and by psychological distress on mitochondrial function and antioxidant response pathways in PBMCs derived from the same cohort of animals, we assessed the level of mitochondrial content (translocase of the outer membrane 20 expression, TOM20) and antioxidant markers (DJ-1, SOD1 and SOD2) by performing Western blot (WB) of lysates from PBMCs derived from WT and PINK1-KO rats that were untreated or exposed to psychological distress. Seven days following stress exposure, WB analyses showed a significantly increase in mitochondrial content in PINK1-KO-PBMCs (increase TOM20, p ≤ 0.01, Fig. 1a) and enhanced antioxidant capacity (increase in the level of SOD2 and DJ1, p ≤ 0.05, Fig. 1b,d). In addition, WT-PBMCs showed a significant decrease in mitochondrial content and SOD2 level following exposure to psychological distress (p ≤ 0.001, Fig. 1).

### Energy metabolism

**Figure 2:**
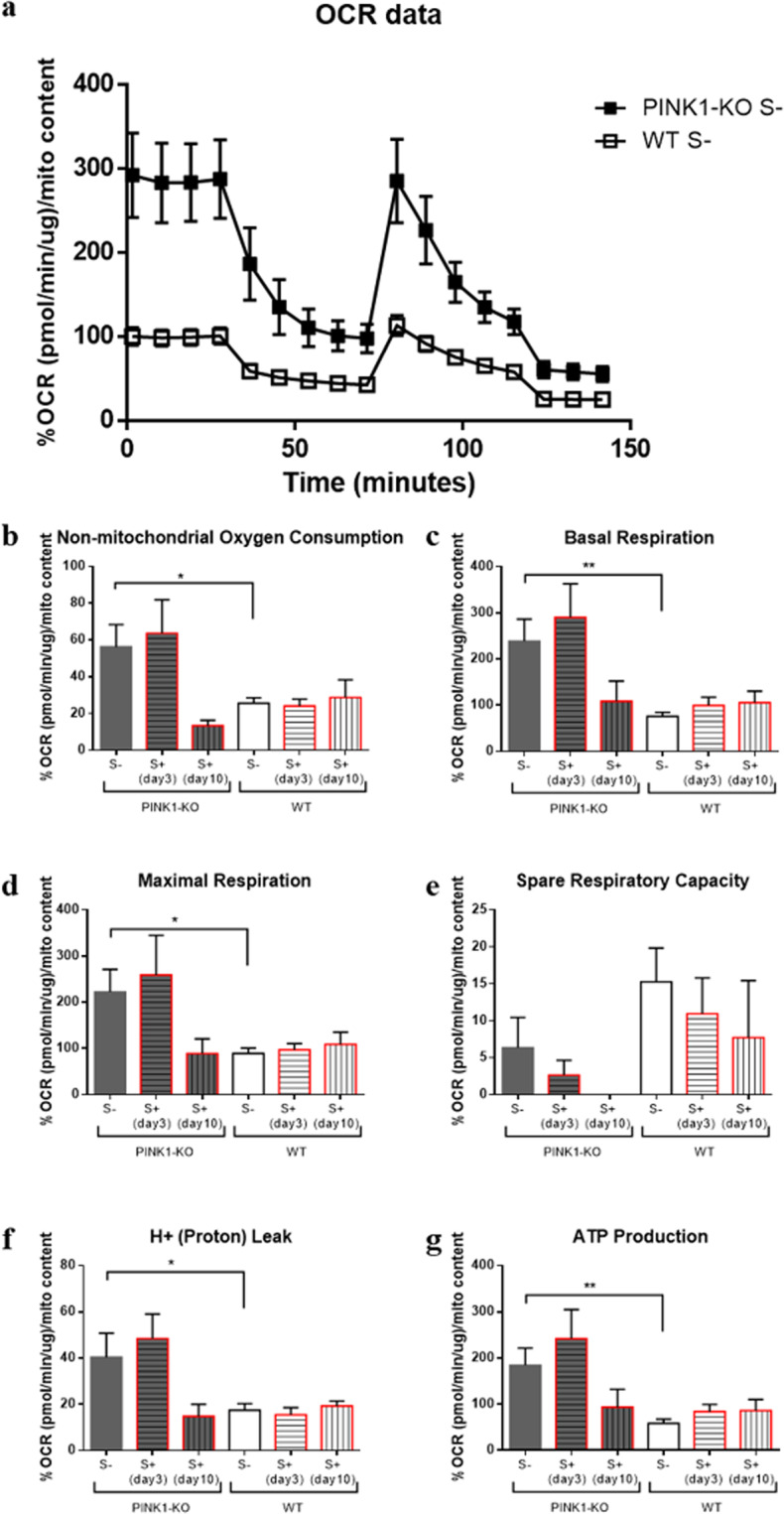
PBMCs show twofold increase in OXPHOS relative to WT-PBMCs. (**a**) OCR data normalized to mitochondrial content for PBMCs derived from unstressed PINK1-KO and WT rats, (**b**) non-mitochondrial oxygen consumption, (**c**) basal respiration, (**d**) maximal respiration, (**e**) spare respiratory capacity, (**f**) H+ (proton) leak, (**g**) ATP production as measured by an XF metabolic bioanalyzer. For each OXPHOS assay, the following compounds were used at the following final concentrations in cells: 1 µM Oligomycin, 1.5 µM carbonyl cyanide 4-(trifluoromethoxy)phenylhydrazone (FCCP), 1 µM Antimycin, 100 nM Rotenone. For control groups (S-), n = 14–15 (5 females and 9–10 males per group) for stressed groups (S+) sacrificed in day 3 the n = 10 (5 females and 5 males per group) and in day 10 the n = 4–5 males per group. OCRs data are expressed as percent means ± SEM relative to time 0 of WT S- group for each cohort/experimental group and normalized to total protein content and mitochondrial content by densitometric analysis of TOM20 levels; *p ≤ 0.05,**p ≤ 0.01, ***p ≤ 0.001, ****p ≤ 0.0001, two-tailed Student’s t-test.

**Figure 3:**
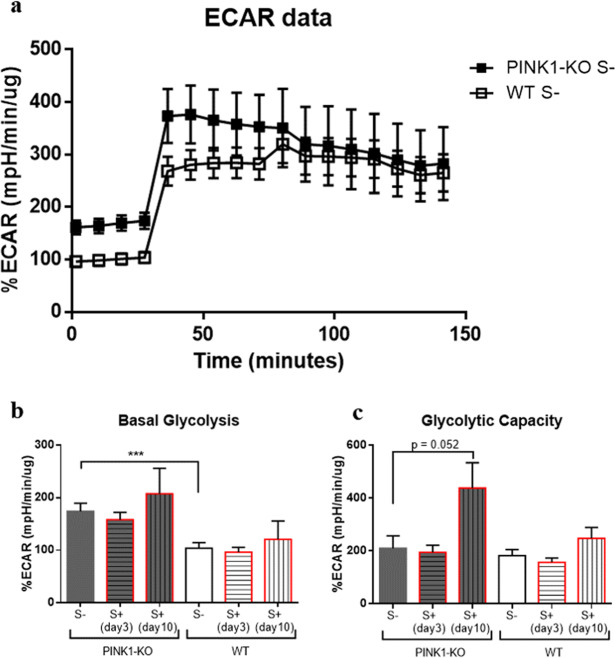
PINK1-KO-PBMCs show a significant increase in basal glycolysis relative to WT-PBMCs whereas glycolytic capacity was increased by psychological distress. (**a**) Extracellular acidification rate **(**ECAR) data analyzed in PBMCs from unstressed PINK1-KO and WT rats, (**b**) basal glycolysis, (**c**) glycolytic capacity. For each ECAR assay, the following compounds were used at the following final concentrations in cells: 1 µM Oligomycin, 1.5 µM FCCP, 1 µM Antimycin, 100 nM Rotenone. For control groups (S-) n = 14–15 (5 females and 9–10 males per group), for stressed groups (S+) sacrificed in day 3 (n = 10, 5 females and 5 males per group), and in day 10 the n = 4–5 males per group. ECAR data are expressed as mean percentage ± SEM relative to time 0 of WT S- group for each respective cohort/experimental condition and normalized to total protein content; *p ≤ 0.05, **p ≤ 0.01, ***p ≤ 0.001, ****p ≤ 0.0001, two-tailed Student’s t-test.

Given that psychological distress significantly disrupts energy metabolism in different brain regions of WT and Parkinsonian rats^3^, we hypothesized that psychological distress alters the bioenergetic state in PBMCs, a well-accepted peripheral tissue used to assess the gradual progression of several chronic diseases and report on the overall health of an animal/human^7,8,55–57^. By employing an XF metabolic bioanalyzer to measure PBMCs bioenergetics (OXPHOS and glycolysis), we observed that PINK1-KO-PBMCs not exposed to stress showed a robust increase in oxygen consumption rates (OCRs) when normalized to protein content (see Supplementary Fig. S2 in the Supplementary Data file), as evidenced by a twofold increase in OXPHOS relative to PBMCs derived from WT rats (WT-PBMCs). Given that long-term psychological distress alters the level of mitochondria in PBMCs (Fig. 1a), we also normalized the OCRs values to the level of mitochondrial content as assessed by measuring the level of the outer mitochondrial membrane protein TOM20 by performing WB analysis (Fig. 2). Hence, by normalizing the OCRs to mitochondrial content, PINK1-KO-PBMCs showed significantly increased non-mitochondrial oxygen consumption (p ≤ 0.05, Fig. 2b), enhanced basal mitochondrial respiration (p ≤ 0.01, Fig. 2c), elevated maximal mitochondrial respiration (p ≤ 0.05, Fig. 2d), increased proton leak (p ≤ 0.05, Fig. 2f) and ATP-dependent OCRs (p ≤ 0.01, Fig. 2g), as well as increased basal glycolysis compared to WT-PBMCs (p ≤ 0.001, Fig. 3b). Consistent with its role in maintaining mitochondrial structure/function, our data suggest that the lack of PINK1, but not exposure to psychological distress, augments OXPHOS and glycolysis in PBMCs. On the other hand, long-term psychological distress (7 days following exposure to psychological distress), diminished the aforementioned OCR parameters in PINK1-KO-PBMCs to a similar extent as WT-PBMCs derived from unstressed animals when normalized to mitochondrial content (Fig. 2b–g) but not when normalized to protein content (see Supplementary Fig. S2 in the Supplementary Data file). These results suggest that psychological distress increases the total amount of mitochondria by two-fold which show a similar level of OXPHOS as WT mitochondria (when OCRs are normalized to mitochondrial content), presumably as a compensatory response to oxidative stress in order to maintain a similar level of energy production. Interestingly, psychological distress did not disrupt the energy metabolism in WT-PBMCs, but it enhanced the glycolytic capacity in PINK1-KO-PBMCs (p = 0.052, Fig. 3c), suggesting that long-term distress alters the mitochondrial metabolism while concomitantly enhancing glycolysis in PINK1-KO-PBMCs.

**Figure 4:**
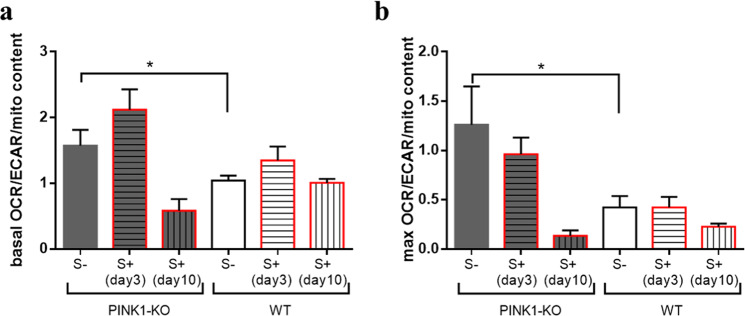
PINK1-KO-PBMCs show higher rate of OXPHOS relative to glycolysis. (**a**) basal OCR/ECAR and (**b**) maximal OCR/ECAR in PINK1-KO and WT rats. OCRs data are expressed as percent means ± SEM relative to time 0 of WT S- group for each cohort/experimental group and normalized to total protein content and mitochondrial content by densitometric analysis of TOM20 levels; ECAR data are expressed as mean percentage ± SEM relative to time 0 of WT S- group for each respective cohort/experimental condition and normalized to total protein content. For control groups (S-), n = 14–15 (5 females and 9–10 males per group), for stressed groups (S+) sacrificed on the 3rd day n = 10 (5 females and 5 males per group), and in day 10 the n = 4–5 males per group. *p ≤ 0.05, **p ≤ 0.01, two-tailed Student’s t-test.

Furthermore, in order to understand which bioenergetic pathway (glycolysis or OXPHOS) is specifically more impacted in PINK1-KO-PBMCs, we plotted the ratio of OCRs to ECARs in basal state (basal OCR/ECAR ratio, Fig. 4a) and when MMP is collapsed by exposure to FCCP (maximal OCR/ECAR, Fig. 4b) for each cohort and experimental condition. Overall, we observed that PINK1-KO-PBMCs modestly relied more on OXPHOS (basal and max OCR/ECAR have values > 1) compared to glycolysis (Fig. 4). Consistent with this observation, WT-PBMCs predominantly relied on glycolysis as principal source of energy when FCCP was injected (Fig. 4b), as reported in previous studies^50,54^. However, PBMCs derived from long-term psychologically distressed PINK1-KO rats shifted fuel utilization to glycolysis, albeit not significantly (p = 0.07, Fig. 4), which did not occur in WT-PBMCs.

**Figure 5:**
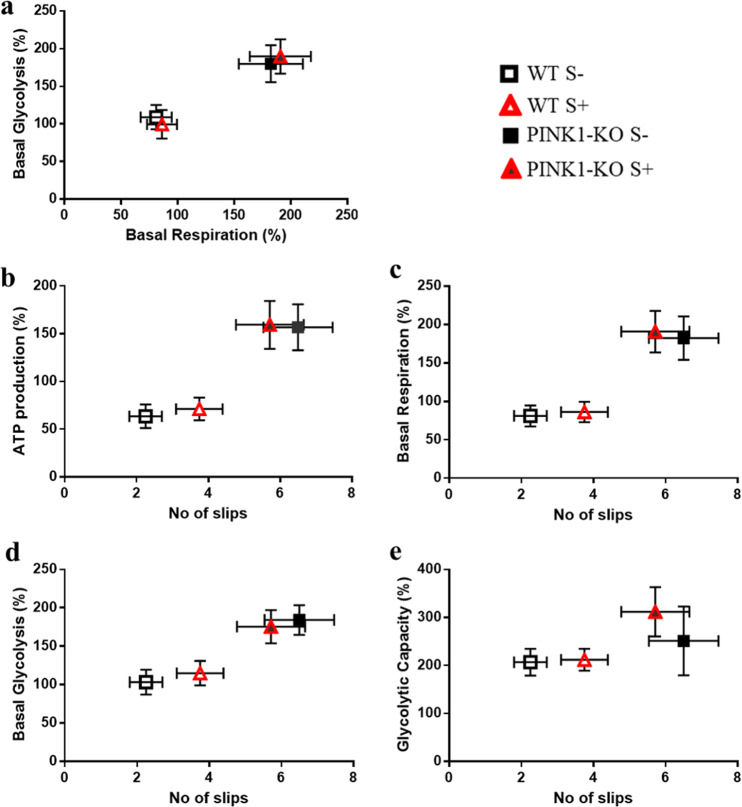
Psychological distress increases the number of slips in WT animals without altering the bioenergetic parameters in PBMCs. Bioenergetic parameters of PBMCs from stressed and unstressed WT rats (WT S + and WT S-, respectively) and from stressed and unstressed PINK1-KO rats (PINK1-KO S + and PINK1-KO S-, respectively) were associated with motor dysfunction as represented by the number of slips (n# of slips, x axis) in the beam balance test in the same cohort of animals. This test was used to measure the fine motor coordination by allowing the animals to cross a 2 m longledge tapered beam at an angle of 15° from the floor. The rats were trained once followed by three trials with 1–2 min of rest between them, as previously described^3^. (**a**) relationship of basal glycolysis vs. basal respiration, (**b**) ATP production vs # of slips, (**c**) basal respiration vs # of slips, (**d**) basal glycolysis vs # of slips and (**e**) glycolytic capacity vs # of slips. Stressed groups represent samples obtained in both day 3 and day 10. OCRs data are expressed as percent means ± SEM relative to time 0 of WT S- group for each cohort/experimental group and normalized to total protein content and mitochondrial content by densitometric analysis of TOM20 levels; ECAR data are expressed as mean percentage ± SEM relative to time 0 of WT S- group for each respective cohort/experimental condition and normalized to total protein content.

Given that OXPHOS and glycolysis are intrinsically interconnected (Warburg effect), we plotted the average basal OCRs relative to the basal ECARs for each animal group as an overall measure of the bioenergetic landscape of PBMCs for each cohort/treatment. In brief, we observed that PINK1-KO-PBMCs showed significantly increased OCRs and ECARs relative to WT-PBMCs, suggesting that a lack of PINK1 enhances the baseline bioenergetic status of those cells. However, psychological distress did not further shift the baseline OCRs or ECARs in PINK1-KO-PBMCs (Fig. 5a).

To correlate the bioenergetic alterations observed in PBMCs with motor symptoms in PINK-KO rats that were untreated or exposed to psychological distress in the same cohort of animals, the different bioenergetic parameters (ATP production, basal respiration, basal glycolysis and glycolytic capacity) analyzed in PBMCs were plotted against the motor symptoms as assed by counting the number of foot slips in the beam balance test as previously reported^3^. In WT animals, exposure to psychological distress nearly doubled the number of foot slips and falls even though ATP-dependent OCRs did not significantly change in PBMCs (Fig. 5b). On the other hand, PINK1-KO rats showed a threefold increase in the number of slips that positively correlated with a 1.5-fold increase in ATP-dependent OCRs and basal respiration production in PINK1-KO-PBMCs relative to WT (Fig. 5b,c, p < 0.01). Thus, the lack of PINK1 leads to bioenergetic alterations in PBMCs which positively correlated with motor symptoms. In addition, the increase in the number of foot slips observed in PINK1-KO rats were also associated with enhanced glycolysis in PINK1-KO-PBMCs (Fig. 5d,e).

### BDNF expression

**Figure 6:**
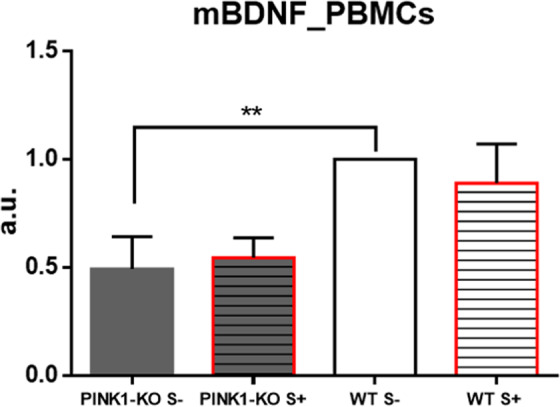
PINK1-KO-PBMCs showed significantly diminished level of BDNF. Densitometric analysis of the mature form of BDNF (mBDNF) for each cohort/treatment were normalized to WT S- group. n = 10 (5 females and 5 males per group). Animals sacrificed immediately stress exposure (day 3). Data are expressed as mean ± SEM; **p ≤ 0.01, two-tailed Student’s t-test.

Given that a decrease in the level of BDNF and other neurotrophic factors have been observed in the postmortem brain tissue and serum from PD patients and in animal models of PD^36–39^, we surmised that the level of BDNF in PBMCs will be decreased as well, which would be consistent with PD pathology. Indeed, PINK1-KO-PBMCs exhibited a significant decrease in the intracellular level of BDNF (p ≤ 0.01, Fig. 6). On the other hand, psychological distress did not alter BDNF level in both WT and PINK1-KO cells. Given that BDNF has been reported to modulate mitochondrial metabolism and functions in leukocytes^45,58,59^, our WB data raises the possibility that the low intracellular level of BDNF observed in PINK1-KO-PBMCs can contribute to reduced mitochondrial function.

## Discussion

The present study sheds light on the vital role that PINK1 plays in modulating the bioenergetic state of PBMCs under physiological conditions and under pathological conditions in the context of oxidative stress induced by psychological distress and in a rat model of PD^3^.

Indeed, PBMCs from young PINK1-KO rats showed a significant two-fold increase in OCRs and basal glycolysis compared to WT rats (Figs. 2–3). In different cell types, previous studies have shown that PINK1 deficiency induces a chronic disruption in mitochondrial metabolism, presumably due to the canonical role of PINK1 in mitophagy regulation and in controlling mitochondrial function and structure^14,16,18,29,60^. Given the critical role of PINK1 in modulating proper calcium handling by mitochondria, a lack of PINK1 elicits a pathological accumulation of calcium in the mitochondrion, which stimulates free radical production that subsequently contributes to mitochondrial dysfunction^61^. In neurons, PINK1 is a cytoprotective serine/threonine kinase as a lack of PINK1 promotes mitochondrial dysfunction, mitochondrial fragmentation and accelerates apoptosis of neurons. However, the molecular mechanisms that underlie the selective vulnerability of neurons relative to other cell types have yet to be elucidated. Indeed, lack of PINK1 function induces differential effects on mitochondrial structure/function in different cell types. For instance, PINK1-KO myocytes showed higher MMP whereas PINK1-KO neurons exhibited a lower MMP relative to WT cells. Moreover, PINK1-KO myocytes showed a higher glycolytic capacity relative to PINK1-KO neurons whereas both cell types exhibited impaired respiration^34^. Like in PINK1-KO cardiac myocytes^34^, our bioenergetic data in PBMCs demonstrated that PINK1-KO-PBMCs showed significantly increased glycolysis. Unlike neurons, these published observations suggest that non-neuronal tissues may show enhanced glycolysis as a compensatory response to mitochondrial dysfunction attributed to a lack of PINK1 function which can confer them the ability to withstand toxic insults compared to neurons.

In our previous study, two and a half months old PINK1-KO rats showed mild motor impairment, no significant alterations in brain energy metabolism and modest but significant neurodegeneration of SN neurons. Interestingly, young PINK1-KO rats showed significant bioenergetic alterations (OXPHOS and glycolysis) in the prefrontal cortex^3^. However, PINK1-KO rats exhibit severe motor deficit and neurodegeneration at 8 months of age^22^. Our study is the first to report metabolic alterations in PBMCs derived from young PINK1-KO rats which coincided with motor deficits, decreased BDNF level, decreased mitochondrial content and antioxidant capacity as observed in midbrain dopamine neurons of the same cohort^3^.

Based on our collective data, we offer the following conceptual model by which PINK1 modulates the bioenergetic state of PBMCs. First of all, the bioenergetic alterations observed in PINK1-KO-PBMCs may be intrinsic (due to mitochondrial dysfunction) or can be induced/exacerbated by external factors (e.g. increased oxidative stress and increased cortisol levels in the bloodstream), phenomena that is associated with neurodegeneration and motor symptoms. The high OCRs and ECARs, as observed in PINK1-KO-PBMCs, suggest an increase in energy demand which is consistent with an overactive immune response, that in turn, can contribute to PD pathology by stimulating chronic inflammation and oxidative stress^46^. However, we recognize that the increase in energy metabolism in PINK1-KO-PBMCs is not specific for PD as it has been observed in other chronic diseases. For instance, an increase in mitochondrially-derived OCRs was observed in PBMCs derived from patients with diabetes mellitus, a phenomenon that was associated with increased oxidative stress^62^. Also, PBMCs from ME/CFS patients showed altered mitochondrial respiration including an increase in ATP production^57^. Similarly, a significantly increased maximal respiration and mitochondrial reserve capacities have been observed in PBMCs derived from patients with autism spectrum disorders^55^. Moreover, we observed a high correlation between the bioenergetic state of PBMCs with motor dysfunction (Fig. 5). Hence, the high energy production in PBMCs can be attributed to the impairment of the neuroimmune axis or a state of high oxidative stress (e.g. disruption of mitochondrion function in neurons and immune cells), which is highly correlated with the development of Parkinsonian-like motor symptoms.

In their resting state, WT-PBMCs rely on both OXPHOS and glycolysis to produce energy, whereas exposure to psychological distress did not alter the bioenergetic state of WT-PBMCs (Fig. 5a). The decrease in the maximal OCR/ECAR ratio (Fig. 5b) suggests that WT-PBMCs preferentially use glycolysis as principal fuel when the proton gradient and the MMP are disrupted by FCCP^53^. This data are consistent with previous studies that demonstrated that basal OXPHOS shifted to glycolysis in PBMCs during an acute infection, presumably as a physiologic mechanism to successfully mount an immunological response^51^. Enhanced glycolysis in aerobic conditions is a hallmark of the inflammatory response, however, this did not occur in PINK1-KO-PBMCs as both OCRs and ECARs equally increased (Fig. 4b). Our data suggest that depletion of PINK1, or lack of PINK1 function, decreases the ability of the immune system to fight against foreign antigens or inflammatory stimuli. It is worth noting that our data are consistent with previous studies that suggest a critical role of PINK1 modulating immune responses^30,31^.

In our recently published study, the energy metabolism in the brain was observed to be adversely affected by psychological distress as both OCRs and ECARs significantly diminished in the prefrontal cortex and concomitantly increased in the SN from both young stressed WT and PINK1-KO rats^3^. Moreover, lipid peroxidation, DNA damage and alterations in protein structure were observed in PBMCs derived from psychologically distressed rats^7,8^. However, psychological distress did not significantly change the overall bioenergetic state of WT-PBMCs whereas PINK1-KO cells exhibited an increase in glycolytic capacity (Fig. 3). Basal OCR/ECAR ratio suggests that PINK1-KO-PBMCs predominantly use OXPHOS relative to WT cells in their resting state (Fig. 4a). However, when mitochondria are treated with respiratory uncouplers (max OCAR/ECAR ratio), stressed PINK1-KO cells modestly shifted from OXPHOS to glycolysis (Fig. 4b), suggesting that depletion of PINK1 decreases the mitochondrion efficiency during oxidative stress elicited by psychological distress. While short term or long-term psychological distress modestly affect the bioenergetic status of PBMCs, our WB data showed that short-term psychological distress significantly decreases the level of mitochondria content and of SOD2 and DJ-1, two key mitochondrial antioxidants. In addition, our WB data suggest that long-term, but not short-term psychological distress, elicited a significant increase in the level of mitochondria (TOM20) and antioxidant capacity (Fig. 1) in PINK1-KO-PBMCs, suggesting that oxidative stress induced by the lack of PINK1 and by psychological distress may stimulate mitochondrial biogenesis and a compensatory increase in mitochondrially-derived antioxidants. However, the increase in the level of mitochondria in PBMCs from psychologically distressed PINK1-KO rats coincided with enhanced glycolysis. Therefore, our data suggest that psychological distress stimulates glycolysis and concomitantly decreases OXPHOS in a larger pool of mitochondria in PINK1-KO-PBMCs. Our compiled bioenergetic data suggest that mitochondria from chronically stressed PINK1-KO-PBMCs are inefficiently coupled compared to unstressed PINK1-KO-PBMCs. Therefore, the observation that the energy metabolism in WT-PBMCs was not affected by psychological distress suggests that depletion of PINK1 would make PBMCs highly vulnerable to oxidative stress, elicits a bioenergetic crisis, and thereby, elicits an increase in the level of antioxidant and energy production pathways, and possibly elicits downstream transcriptional activation of survival and antioxidant gene programs in a similar manner as induced by tumors^63^. In addition, based on available bioenergetic data, the percentage of active mitochondria, between WT and PINK1-KO and before/after psychological distress, is likely similar for all of the various experimental groups and conditions, given that the percentage of OCR required to produce ATP (ATP-dependent OCR) in PINK1-KO-PBMCs is approximately 40% higher as in WT-PBMCs (Fig. 2), regardless of whether animals were exposed to psychological distress. While our compiled data suggest that the lack of PINK1 and the exposure to psychological distress may adversely affect the bioenergetic status and antioxidant responses in PBMCs (Figs. 2–5), additional studies that interrogate the redox status (level of ROS species) and transmembrane potential of mitochondria in PBMCs are needed to confirm these observations. Nonetheless, this study paves the way for performing additional experiments to gain more knowledge on specific redox species produced by PBMCs from WT vs. PINK1-KO animals exposed to psychological distress. Although we cannot ascertain with accuracy when oxidative stress (e.g. increased superoxide, hydrogen peroxide, and hydroxyl radicals) is enhanced in PBMCs from animals exposed to psychological distress, we have previously published that psychological distress induces significant lipid, protein and DNA oxidation analyzed by SR-µFTIR^7,8^, in PBMCs derived from WT rats after five days of stress exposure.

Additionally, PINK1-KO-PBMCs showed altered levels of BDNF. The level of intracellular BDNF was observed to be significantly diminished in PINK1-KO-PBMCs relative to WT-PBMCs (Fig. 6). Consistent with our data obtained in PBMCs, the level of intracellular BDNF was observed to be significantly reduced in the midbrain of the same cohort of young PINK1-KO rats that exhibited motor disfunction and increased anxiety^3^. However, psychological distress did not significantly affect the intracellular level of BDNF in PBMCs suggesting that oxidative stress induced by psychological distress causes differential effects on the level of BDNF in the brain and peripheral tissues. BDNF is involved in maintaining neuronal homeostasis of neurons and in regulating select physiological functions in immune cells. Indeed, previous studies have shown that this neurotrophic factor regulates the neuroimmune axis, and modulates signaling pathways involved in cell survival, growth and function (e.g. Ras-PI3K-Akt and Ras-MAPK-Erk)^64^. BDNF is critical for neuronal homeostasis as altered levels of BDNF in postmortem brain tissue, PBMCs, serum or plasma from humans have been correlated with the onset of major clinical depression and other mood disorders^58,64^ or PD^36–41,65,66^. PBMCs are an important source of BDNF and express high levels of its cognate receptor, the tyrosine protein kinase receptor B (TrkB). Recent studies have shown that BDNF is critical for modulating the immune system as the binding of BDNF to TrkB activates PBMCs^45,58,59^ and modulates autocrine and paracrine signaling that connects the nervous and immune systems^67^. In addition, BDNF has a prominent autocrine function in B cells as it plays a vital role in the regulation of apoptotic pathways under conditions of high oxidative stress^68^. This neurotrophic factor is considered a potential therapeutic marker for mood disorders, neurodegenerative diseases or brain injuries^64,69^, and is elevated during periods of intense physical exercise^70^. A decrease in the level of BDNF in PBMCs is associated with neuropathology in various chronic, neuroimmune disorders including multiple sclerosis^71^ or in brain inflammation^45^. Interestingly, some antidepressant drugs^66^ modulate and improve the level of BDNF in plasma and immune cells.

## Conclusions

Although the involvement of PINK1 in neurodegeneration has been extensively studied in the brain, it is still unclear what physiological roles PINK1 plays in peripheral tissues and the extent to which it intersects with the neuroimmune axis. The present study suggests that PINK1 plays a crucial role in modulating the bioenergetic state of PBMCs under basal conditions, and in the context of increased energy demand and oxidative stress. Unexpectedly, the depletion of PINK1 induces a significant increase in energy consumption in PBMCs (OXPHOS and glycolysis) and a concomitant decrease in the level of neurotrophic factors (e.g. BDNF) and antioxidant response pathways. PBMCs derived from stressed animals exhibited an over-reliance on glycolysis to compensate for possible mitochondrial dysfunction caused by a lack of PINK1 and by psychological distress. Although we recognize that more studies need to be performed in additional animal cohorts and subsequently corroborated in humans, our study suggest that monitoring both the bioenergetics and level of BDNF in PBMCs can serve as valuable tool for monitoring overall health in peripheral tissues and oxidative stress induced by psychological distress.

## Methods

### Study design

**Figure 7:**
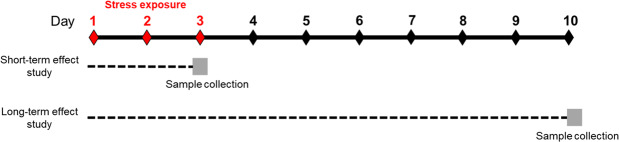
Study design. Animals were stressed for three consecutive days using a predator-induced psychological distress model (from day 1 to day 3). Two cohorts of animals were used to study the effects of psychological distress on PBMCs bioenergetics. The first cohort comprised of 10 WT and 10 PINK1-KO females, and 10 WT and 10 PINK1-KO males were employed to study the short-term effect of distress, after which, animals were sacrificed immediately after stress exposure on day 3. The second cohort conformed by 8 WT and 10 PINK1-KO males, was used to study the long-term effect of distress and the animals were sacrificed on day 10.

To study the effects of psychological distress on the bioenergetics and mitochondrial health in PBMCs, young wild type (WT) and Parkinsonian rats (PINK1-KO) were stressed for three consecutive days by using the predator-induced psychological distress model as previously described^3^. In brief, two cohorts of animals were used: one to study the effects of acute distress or short-term effect study (animals were sacrificed immediately after stress exposure, in the third day), comprised of 10 WT and 10 PINK1-KO females and 10 WT and 10 PINK1-KO males; and another cohort was employed to study the effects of long-term psychological distress on the bioenergetics of PBMCs (sacrificed seven days following stress exposure, in the day 10), formed by 8 WT and 10 PINK1-KO males (Fig. 7). Finally, six experimental groups were used: WT unstressed rats (WT S-), WT stressed rats (WT S+) sacrificed in day 3, WT stressed rats sacrificed in day 10, PINK1-KO unstressed rats (PINK1-KO S-), PINK1-KO stressed rats (PINK1-KO S+) sacrificed in day 3, PINK1-KO stressed rats sacrificed in day 10. To facilitate the interpretation of the data, control groups (WT S- and PINK1-KO S-) results from acute and long-term assays were put together and the final number of animals in each groups were: for the WT S-, n = 14 rats, for WT S+ sacrificed in day 3, n = 5, for WT S+ sacrificed in day 10, n = 4, for PINK1-KO S- n = 15, for PINK1-KO S+ sacrificed in day 3, n = 5, for PINK1-KO S+ sacrificed in day 10, n = 5.

An XF metabolic bioanalyzer was employed to analyze the bioenergetic profile (OXPHOS and glycolysis) of PBMCs derived from unstressed and stressed WT or PINK1-KO rats. For details, see below in the “Analysis of OXPHOS and glycolysis” of the Materials/Methods section. In addition, to correlate bioenergetic alterations with changes in the level of key mitochondrial-localized molecular players involved in modulating antioxidant response pathways and OXPHOS, Western blot (WB) was performed to analyze the level of antioxidants proteins in cell lysates from PBMCs including cytosolic superoxide dismutase (SOD1), mitochondrial superoxide dismutase (SOD2), DJ-1, mitochondrial content (TOM20), and brain-derived neurotrophic factor (BDNF) expression.

To study the effect of acute psychological distress on the bioenergetics of PBMCs, we employed 10 WT and 10 PINK1-KO females, and 10 WT and 10 PINK1-KO males. To study the effects of long-term effects of psychological distress on the bioenergetics of PBMCs, we employed 8 WT and 10 PINK1-KO males. Due to the fact that no gender-associated differences were observe in the aforementioned proteins of interest, all the data were compiled for both genders for each cohort and experimental treatments for the purposes of performing statistical analysis.

### Animals

Long-Evans male and female rats were used for both WT and PINK1-KO (Horizon Discovery), 9 to 11 weeks of age, and weighting 250 g to 350 g. The animals were individually caged three days before the experiments, maintained under controlled temperature 25–26 °C, 12/12 h light/dark cycle, with food and water *ad libitum*.

All animal experiments were performed according the Animal Research: Reporting of *In Vivo* Experiments guidelines^72^ and by using an animal protocol (#572) that was approved by the Institutional Care and Use Committee at the University of Nevada, Reno, US.

### Psychological distress induction

As previously published, between 9 to 11 am, the rats were stressed by being exposed to cat urine for three days^3,7^. Briefly, the animals were habituated for 20 min into the test cage, after which were returned to the housing cage for other 20 min to rest, and finally, the animals were transferred again in a test cage where a urine-impregnated piece of cloth was hanged. The cat urine was pooled from the bladder of three different cats. Approximately 0.5 mL of it was dispensed on each cotton cloth and finally stored at −20 °C until used^7^.

### Isolation of PBMCs

Rats were anesthetized by inhalation of 5% isoflurane. Blood was then extracted via cardiac puncture by using vacutainer tubes containing 7.2 mg K_2_EDTA. Blood was then diluted with 1X PBS (137 mM NaCl, 8.2 mM Na_2_HPO_4_, 1.5 mM KH_2_PO_4_, 3.2 mM KCl, pH 7.4) and slowly transferred onto a bed of 60% Percoll solution. Following centrifugation at 1,000 × g for 30 min at 30 °C, the PBMCs in the pellet were saved and washed two times (600 × g for 10 min) with 1X PBS^7^ supplemented with 25 mM glucose and 0.1% BSA. The cell pellet was then resuspended in warm DMEM supplemented with 10 % FBS and 2 mM Glutamine.

### Analysis of oxidative phosphorylation (OXPHOS) and glycolysis

The levels of OXPHOS and glycolysis in PBMCs derived from unstressed and stressed WT and PINK1-KO rats were measured by employing a Seahorse XF24 Extracellular Flux Analyzer (Agilent, Santa Clara, CA) as previously published^73,74^, but with the following minor modifications. In brief, the real-time of oxygen consumption rates (OCRs), which is an indicator of OXPHOS, and extracellular acidification rates (ECARs), which is an indirect measurement of glycolysis, were monitored by quantifying the following parameters: the non-mitochondrial oxygen consumption (the OCRs measured following injection with rotenone and antimycin-A), the basal respiration (the last OCR measured prior to exposing cells to the ATP synthase inhibitor oligomycin), the maximal respiration (the maximum OCR rate measurement obtained following exposure of cells with the mitochondrial uncoupler FCCP), the proton (H^+^) leak (the residual OCRs measured following oligomycin injection minus the OCRs obtained following rotenone/antimycin A injection), the ATP production (the baseline OCRs minus the OCRs obtained following exposing cells to oligomycin injection), and the mitochondrial reserve capacity (the maximal OCR minus the basal OCR). In addition, the following ECARs parameters were evaluated: basal glycolysis (the last ECAR measured before oligomycin injection), and the glycolytic capacity (the difference between maximum ECARs following FCCP injection and the last rate measurement before oligomycin injection). Also, the basal OCR/ECAR ratio (the ratio between the average of the all points from baseline OCRs measurement prior to the first injection and the average of the all points from ECARs measurement prior to the first injection) and maximal OCR/ECAR ratio (the ratio between the average of the all points from OCRs measured after FCCP injection and the average of the all points from ECARs measurement after FCCP injection prior to the rotenone and antimycin-A injection) were calculated.

After PBMCs were isolated, cells were counted, and a dilution was made to give a final concentration of 7 ×10^6^ cells per mL which were suspended in pre-warmed 37 °C DMEM supplemented with 10 % FBS and 2 mM L-glutamine. Next, 7 ×10^5^ cells in 100 µL were plated into each well of a 24-well XF cell culture microplate. Cells were plated in quintuplicates per animal for up to 4 animals per condition. Following a 2 h of incubation at 37 °C with 5% CO_2_, cells were attached to the bottom of the wells and washed twice with warm XF medium supplemented with 25 mM glucose, 2 mM L-glutamine and 1 mM sodium pyruvate, pH 7.4. The cells were then incubated for an hour at 37 °C, in a non-CO_2_ incubator, in a final volume of 630 µL. Further, drugs were injected by the extracellular flux analyzer and cells in each well were exposed to a final concentration of 1 µM oligomycin (1404-19-9, Sigma Aldrich), 1.5 µM carbonyl cyanide 4-(trifluoromethoxy) phenylhydrazone (FCCP, 370-86-86, Sigma Aldrich), 1 µM antimycin-A (1397- 94-0, Sigma Aldrich) and 100 nM rotenone (R8875, Sigma Aldrich) in a final volume in each well of 860 µL as previously published^73^. Four blank wells for each microplate were used to assess for background levels of OCRs and ECARs. To evaluate the baseline OCRs in the XF metabolic bioanalyzer, four cycles were evaluated, up to five cycles were employed to evaluate ATP-dependent OCRs, five cycles were used to assess for maximal OCRs whereas three cycles were used to measure for mitochondrial-dependent OCRs. The measuring cycles were programmed to consist of 3 min of mixing, 3 min of waiting, and 2 min of measuring OCRs and ECARs. Please note that OCRs and ECARs data were normalized to protein concentration (using Bradford method for protein quantification^75^) as pmol of O_2_/min/μg and mpH/min/μg, respectively, as previously published^3^. Moreover, the OCRs data were normalized to mitochondrial content by densitometric analysis of TOM20 levels obtained using WB assay.

### Western Blot (WB) assays

PBMCs derived from WT or PINK1-KO rats that were untreated or exposed to a psychological distress were homogenized in cold RIPA buffer (150 mM NaCl, 1 % Triton X-100, 0.5 % Sodium Deoxycholate, 0.1 % Sodium Dodecyl Sulfate, 50 mM Tris Base, 2 mM Phenylmethylsulphonyl fluoride, pH 8.0) and centrifuged for 20 min at 19,000 x g, at 4 °C. Approximately 25–30 μg of cell lysates were electrophoresed on 12 % SDS-PAGE gels at 100 volts by using the Bio-Rad minigel system (Hercules, CA, USA). The proteins contained within the SDS-PAGE gels were then transferred from to PVDF membranes, at 15 V for 1 h, using a Bio-Rad semi-dry transfer apparatus (Hercules, CA, USA). In brief, the PDVF membranes were blocked in 4 % milk in 1X TBST (19.8 mM Tris, 150 mM NaCl, 0.05 % Tween-20, pH 7.6) for 1 h, and then were incubated for at least 12 h with the following primary human antibodies targeting the following antigens at 4 °C: SOD1 (Abcam, ab16831, 1:2000, 17 kDa), SOD2 (Abcam, ab13533, 1:5000, 25 kDa), DJ-1 (Abcam, ab76008, 1:5000, 23 kDa), BDNF (Abcam, ab108319, 1:500, 14 and 28 kDa), TOM20 (Santa Cruz, sc11415, 1:500, 17 kDa) or β-tubulin (Abcam, ab131205, 1:5000, 50 kDa). The PDVF membranes were then washed extensively in 1X TBST (5–10 min per washed) and incubated with the respective horse radish peroxidase (HRP)-conjugated secondary antibodies for at least 2 h at room temperature (25 °C): Goat anti-Rabbit IgG (Invitrogen, 65–6120, 1:5000) and Goat anti-Mouse IgG (Invitrogen, 62–6520, 1:5000), and finally analyzed by chemiluminescence detection (ChemiDoc MP Imaging System 170–8280, Bio-Rad, Hercules, CA, USA) as previously published^3^.

Densitometric analysis were performed by using NIH ImageJ 1.50i software (Bethesda, MD) and the integrated density for each immunoreactive band of interest was normalized to β-tubulin and corrected for background by using a rolling ball radius of 25 pixels^3,15^.

### Statistical analyses

The metabolic data was expressed as the mean percentage ± SEM relative to WT without stress group (WT S-). WB data were expressed as the mean ± SEM arbitrary units relative to WT S- group. GraphPad Prism software (version 6.0) was used for Student’s t test (two-tailed) analysis, *p* values fewer than 0.05 were considered statistically significant.

## Supplementary information

Supplementary data.

## Data Availability

All data of this study are available from the corresponding authors.
