# Supplementary material for: Psychological distress and lack of PINK1 promote bioenergetics alterations in peripheral blood mononuclear cells
Source: Sci Rep. 2020 Jun 17;10:9820. doi: 10.1038/s41598-020-66745-9 (PMC7300038; doi:10.1038/s41598-020-66745-9)

Psychological distress and lack of PINK1 promote bioenergetics alterations in peripheral blood mononuclear cells

Grigoruţă, Mariana, Dagda, Ruben K[*], Díaz-Sánchez, Ángel G., Martínez-Martínez, Alejandro[*]

*To whom correspondence should be addressed to:
Alejandro Martínez-Martínez; Email: alejandro.martinez@uacj.mx;
Dagda, Ruben K.; Email: rdagda@med.unr.edu;

Supplementary Figure S1

We attached raw images of Western blots performed to analyze the levels of antioxidants DJ1 (23 kDa), TOM20 (17 kDa), SOD1 (17 kDa), SOD2 (24 kDa) and BDNF (14 and 28 kDa) expression in PBMCs derived from stressed and unstressed WT and PINK1-KO rats.

Please note that separate gels were preformed for TOM20 and DJ1 study from SOD1, SOD2 and BDNF to avoid overlapping of the bands, as proteins have approximately same molecular weight. β-tubulin (50 kDa) was used as internal control.

Each blot was cut in three parts under 50 kDa (for β-tubulin) and under 20 kDa (for SOD2/ DJ1/ mBDNF) to separate the membrane where TOM20 or SOD1 are expressed. Each membrane was separately incubated with the corresponding antibodies.

For the data analysis, we have excluded 4 Western blot lanes that showed little to no β-tubulin immunoreactivity.

Supplementary Figure S1

MALES - Acute effect

Blot 1 for DJ1 and TOM20

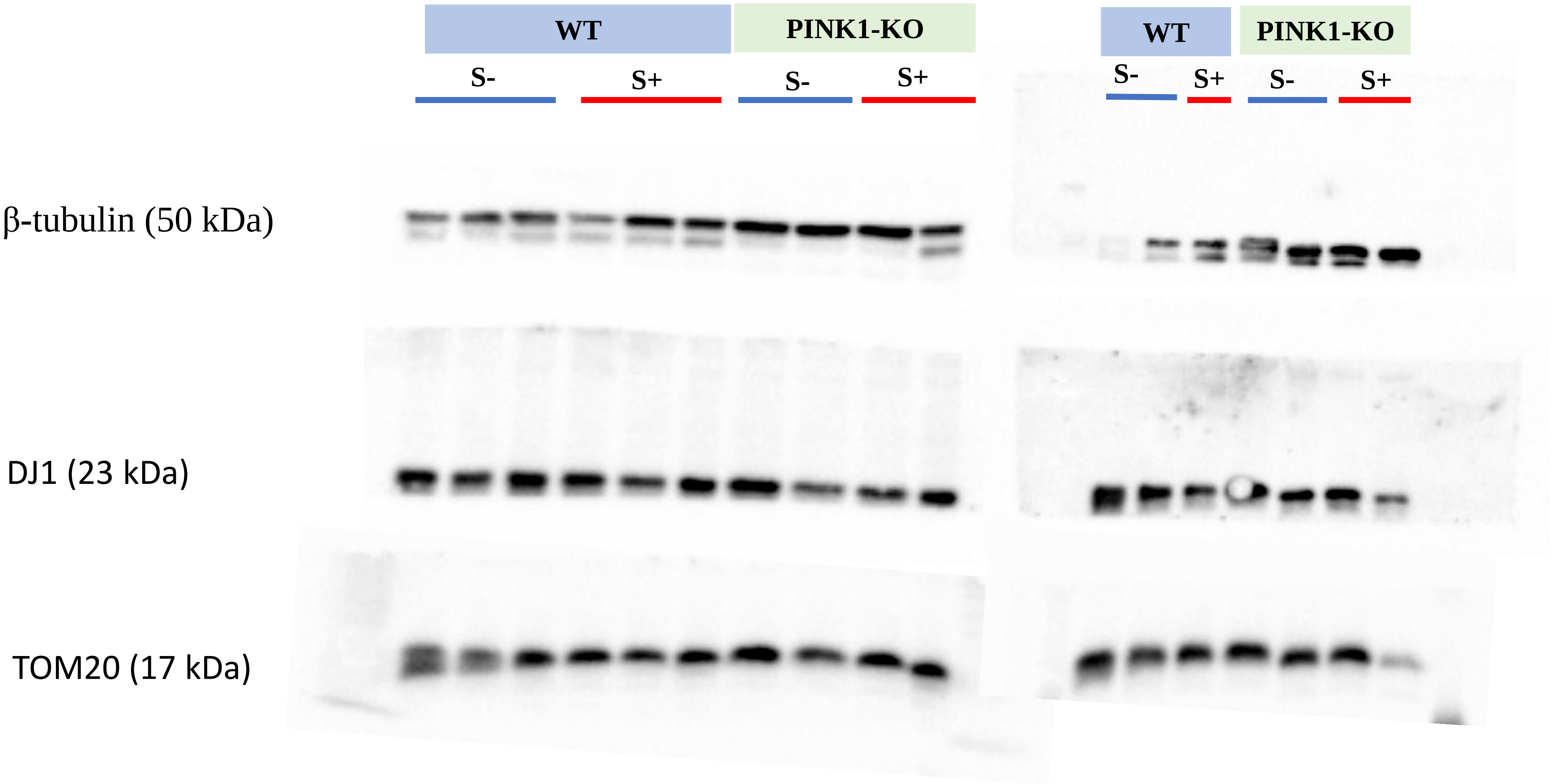

Supplementary Figure S1

MALES - Acute effect

Blot 2 for DJ1 and TOM20

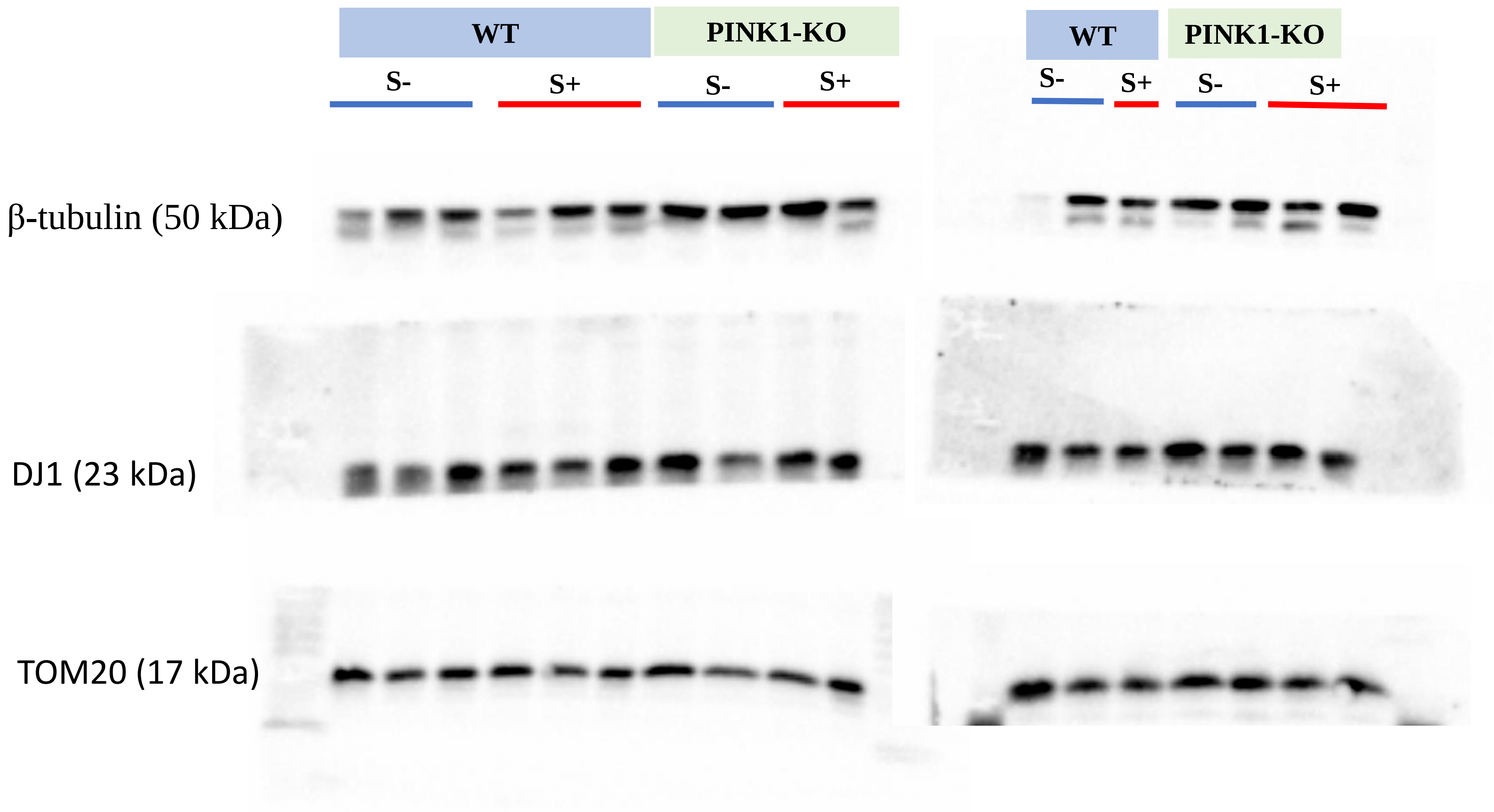

Supplementary Figure S1

MALES - Acute effect

Blot 1 for SOD1, SOD2, BDNF

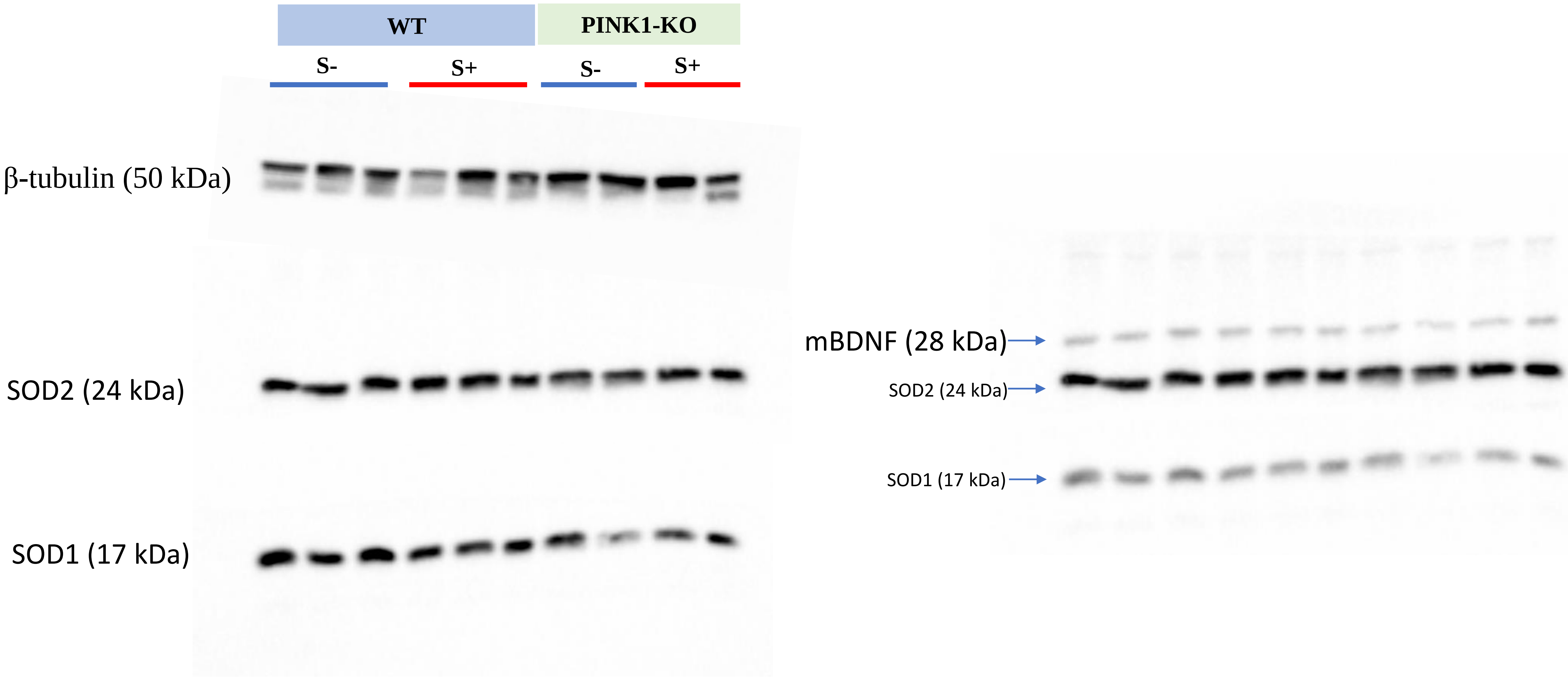

Supplementary Figure S1

MALES - Acute effect

Blot 1 for SOD1, SOD2, BDNF

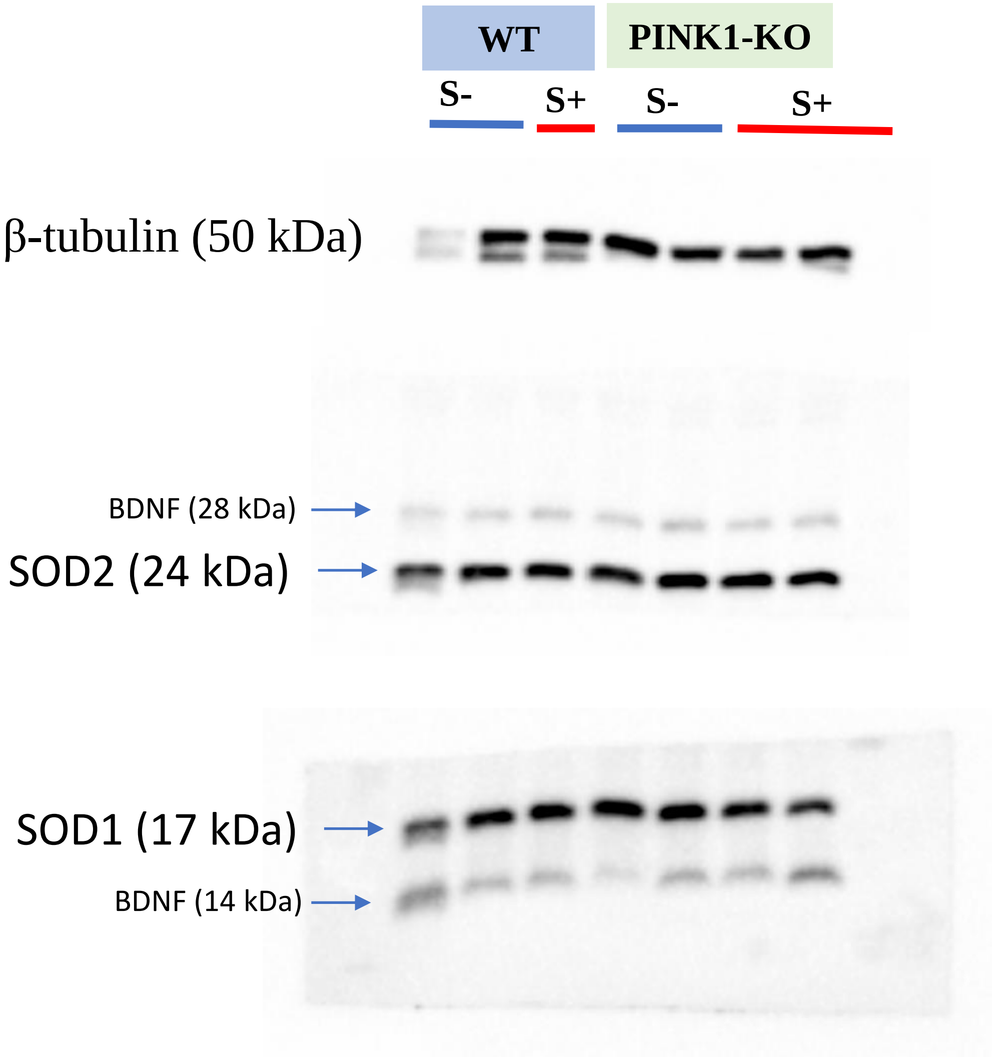

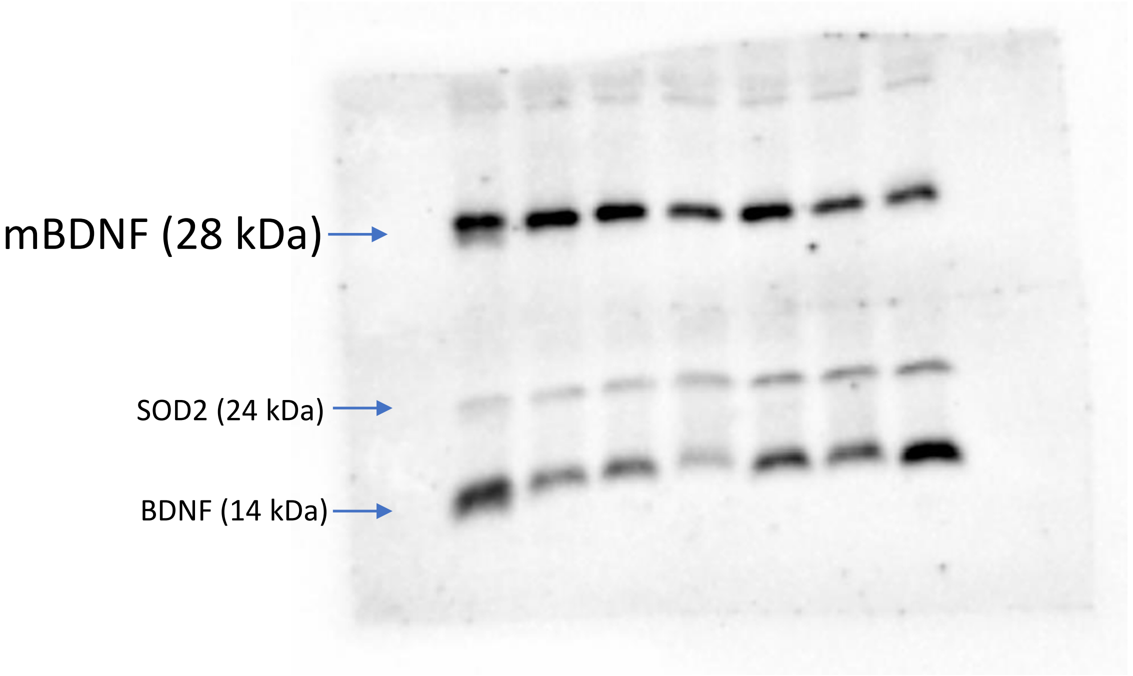

Supplementary Figure S1

MALES - Acute effect

Blot 2 for SOD1, SOD2, BDNF

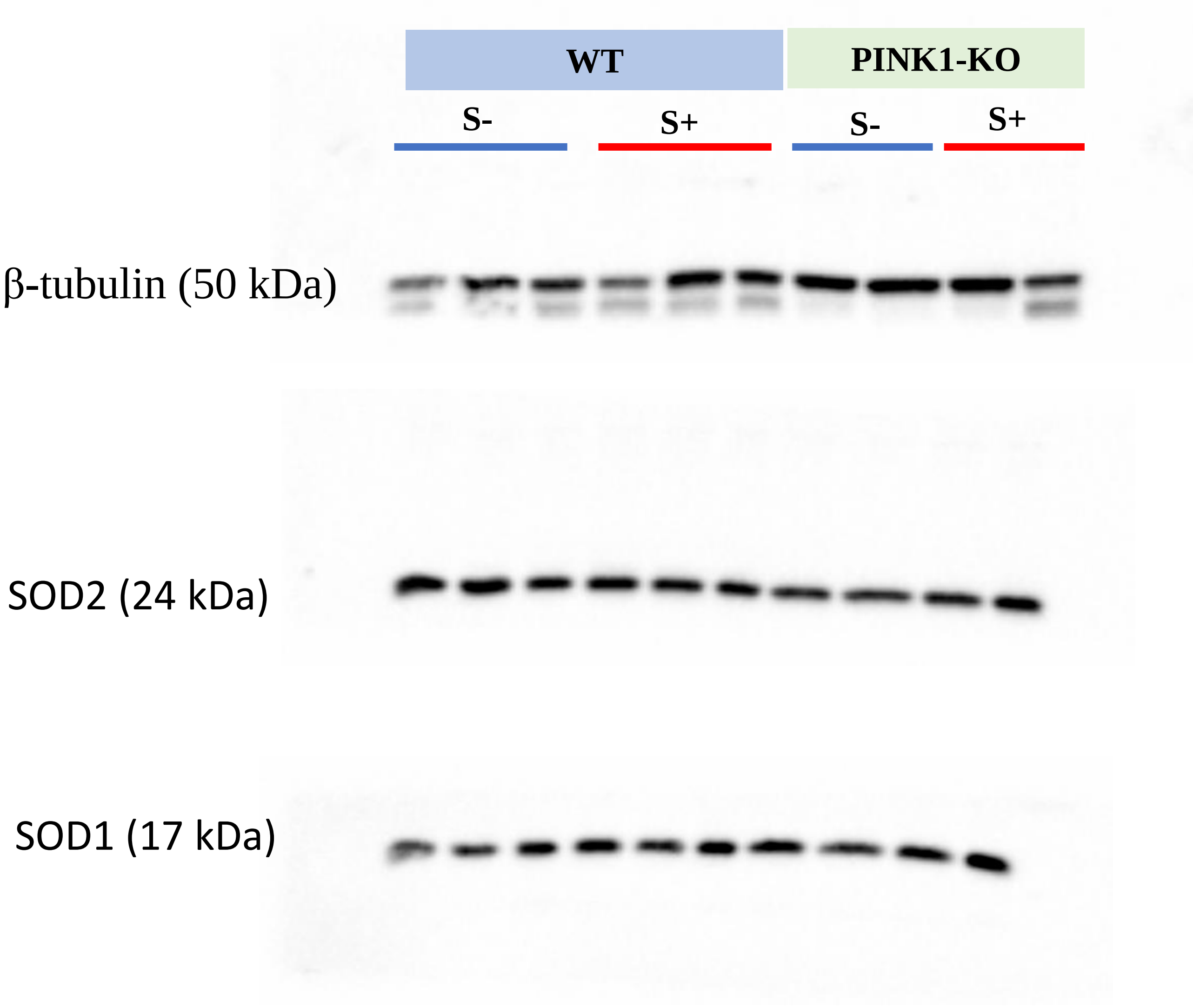

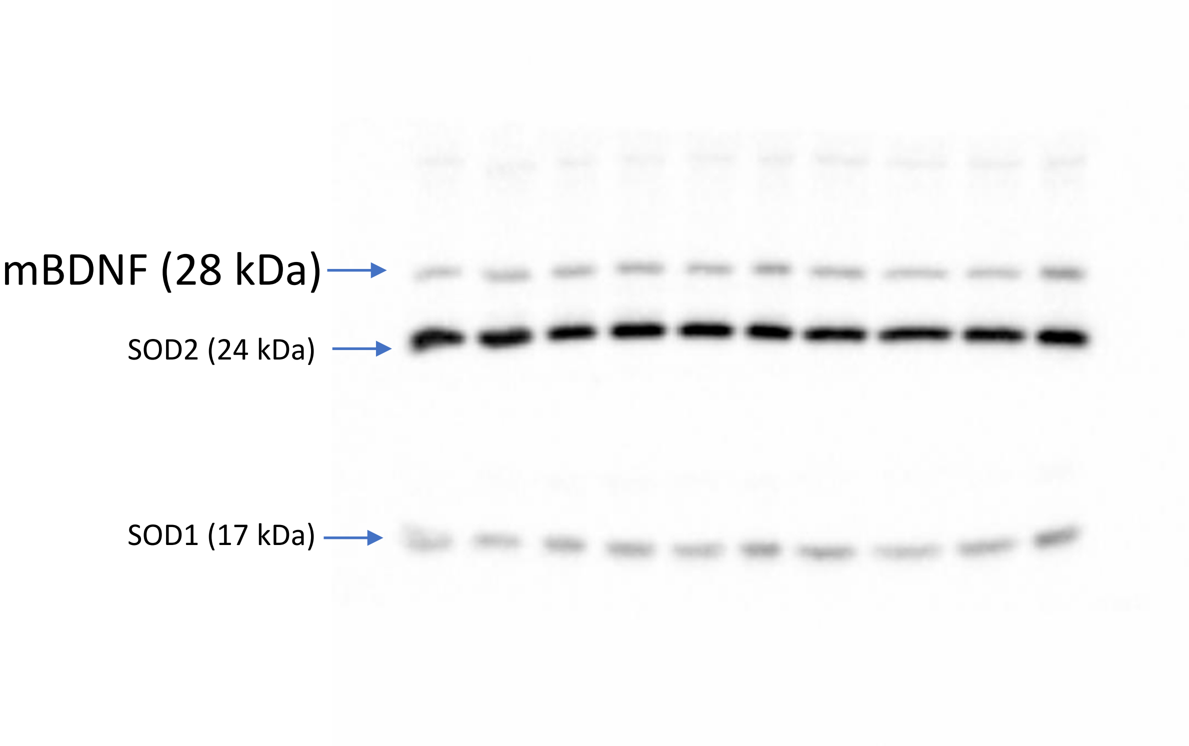

Supplementary Figure S1

MALES - Acute effect
Blot 2 for SOD1, SOD2, BDNF

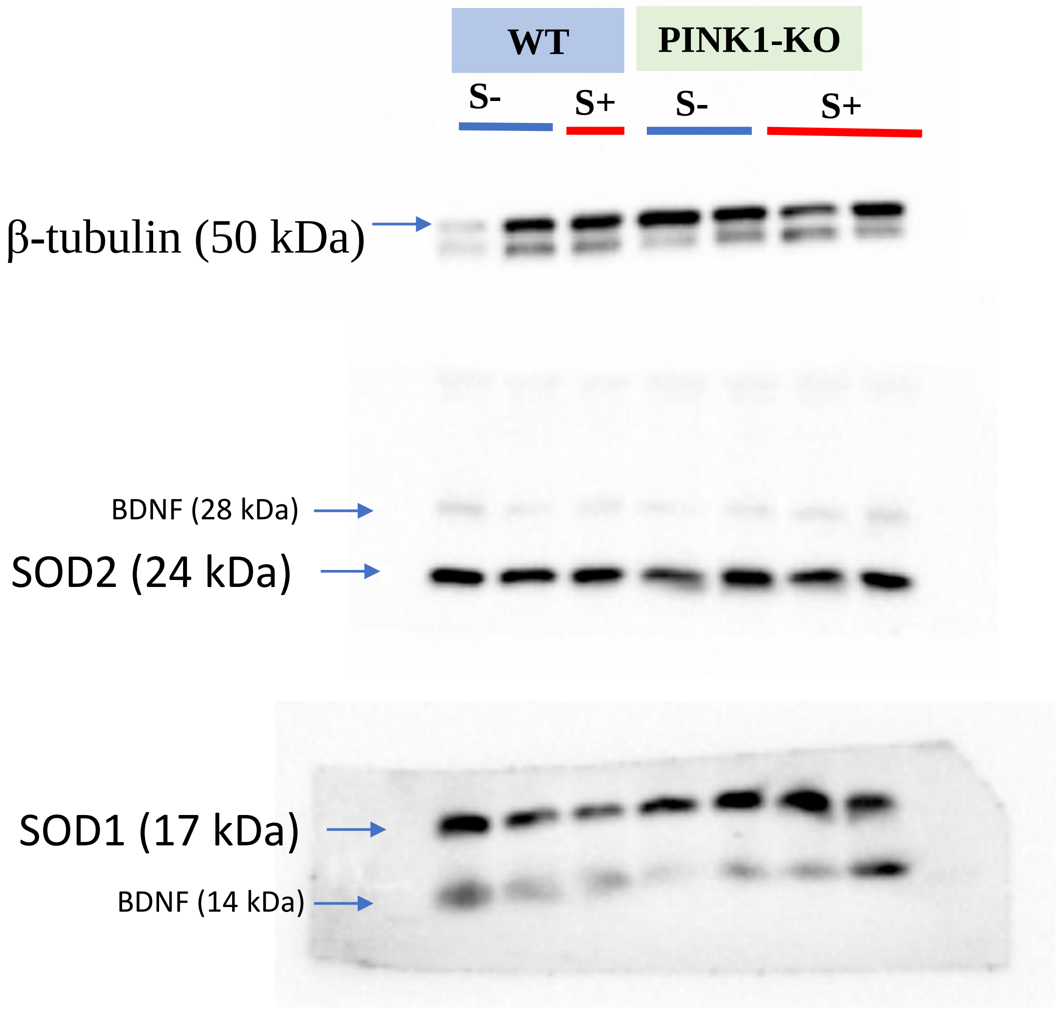

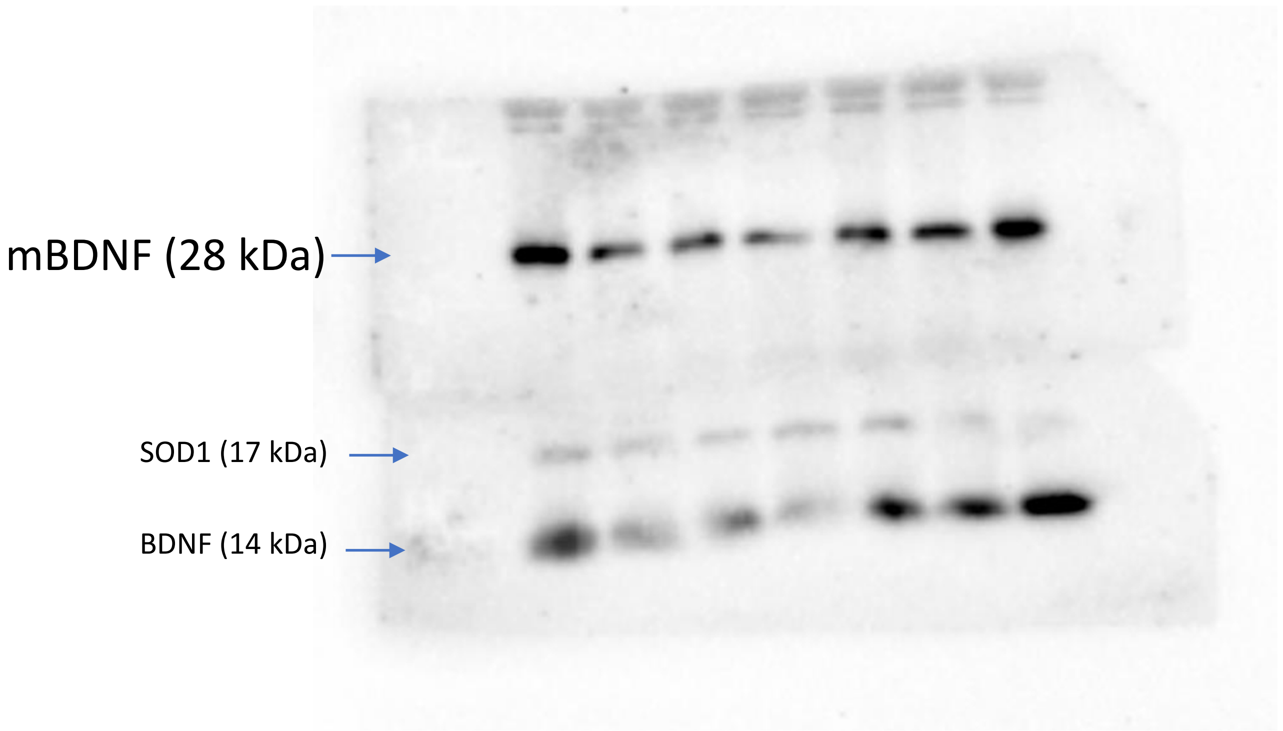

Supplementary Figure S1

MALES - Long-term effect
Blot 1 for DJ1 and TOM20

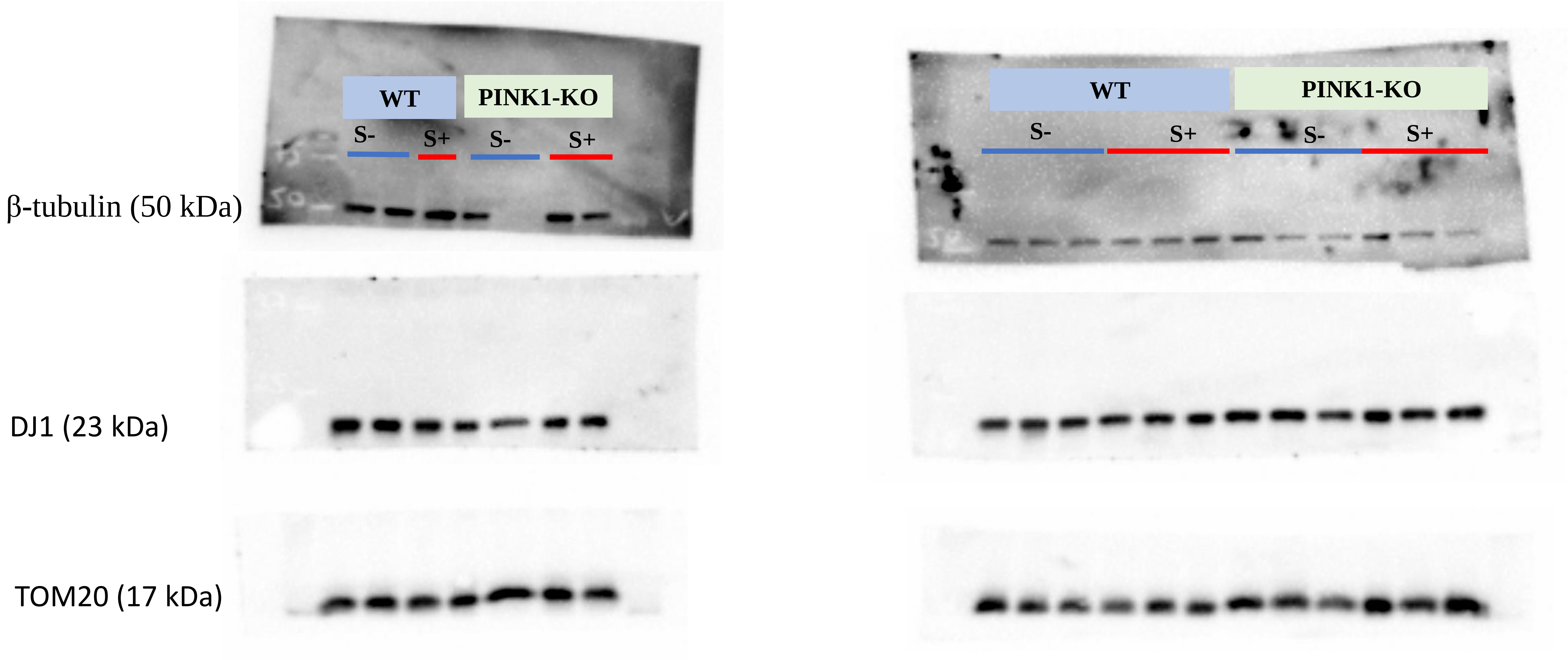

Supplementary Figure S1

MALES - Long-term effect

Blot 2 for DJ1 and TOM20

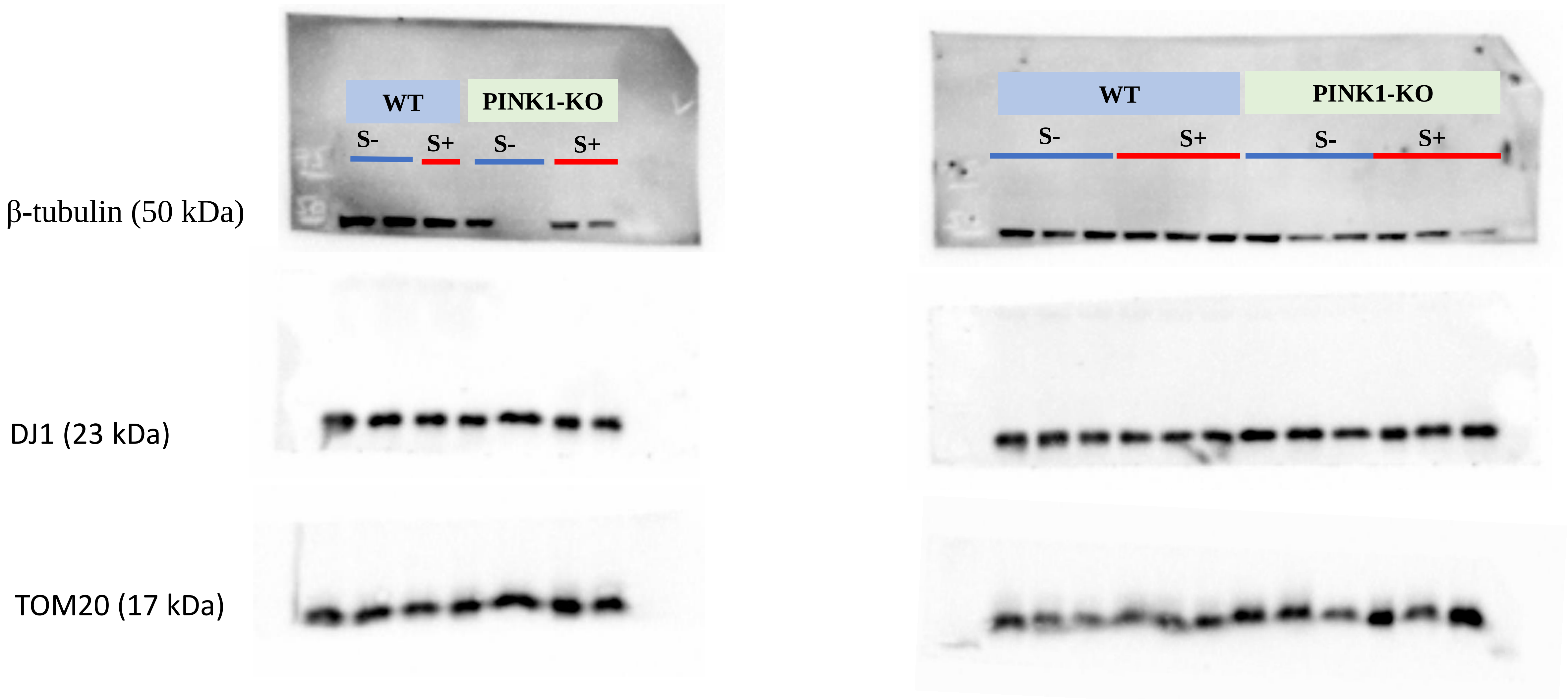

Supplementary Figure S1

MALES - Long-term effect

Blot 1 for SOD1 and SOD2

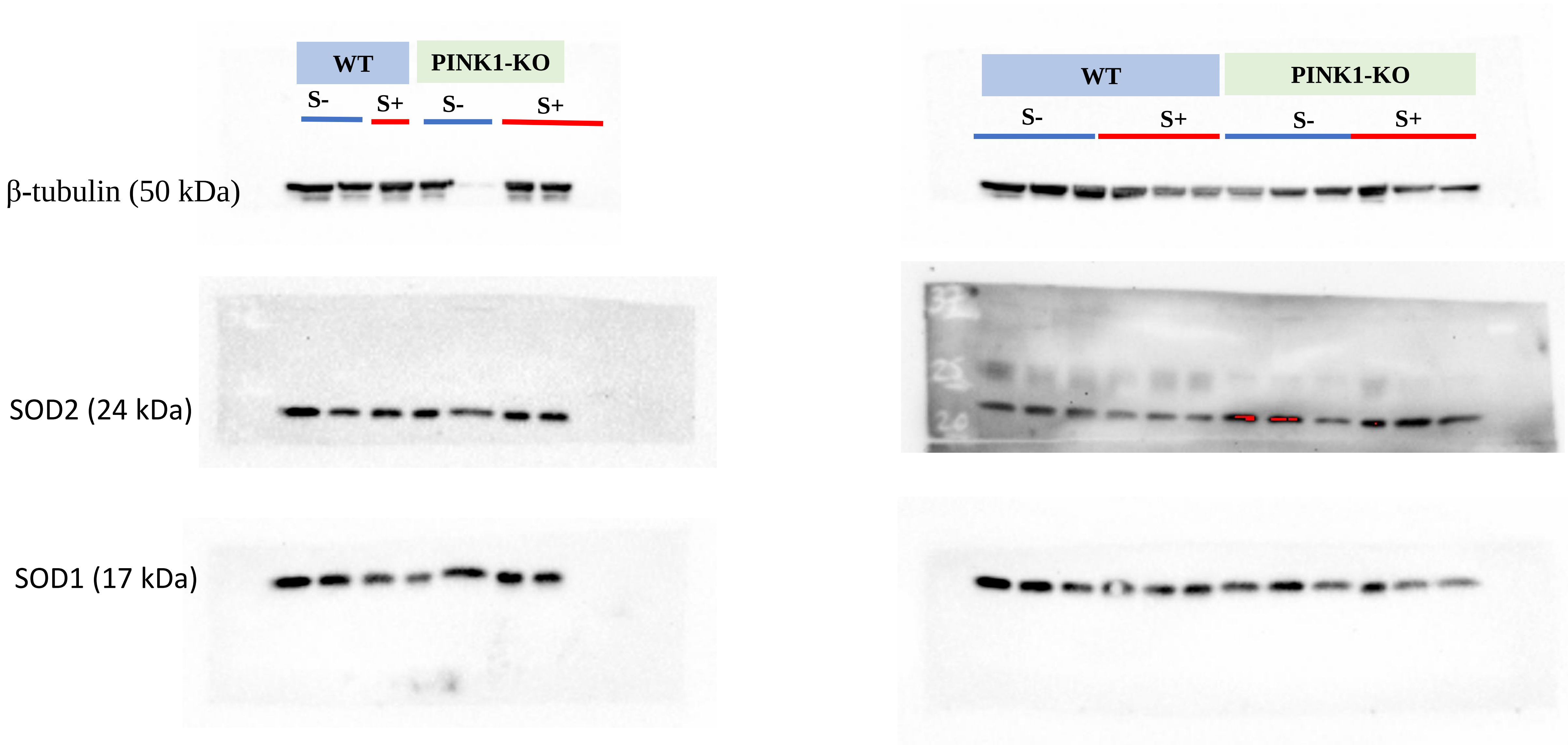

Supplementary Figure S1

MALES - Long-term effect

Blot 2 for SOD1 and SOD2

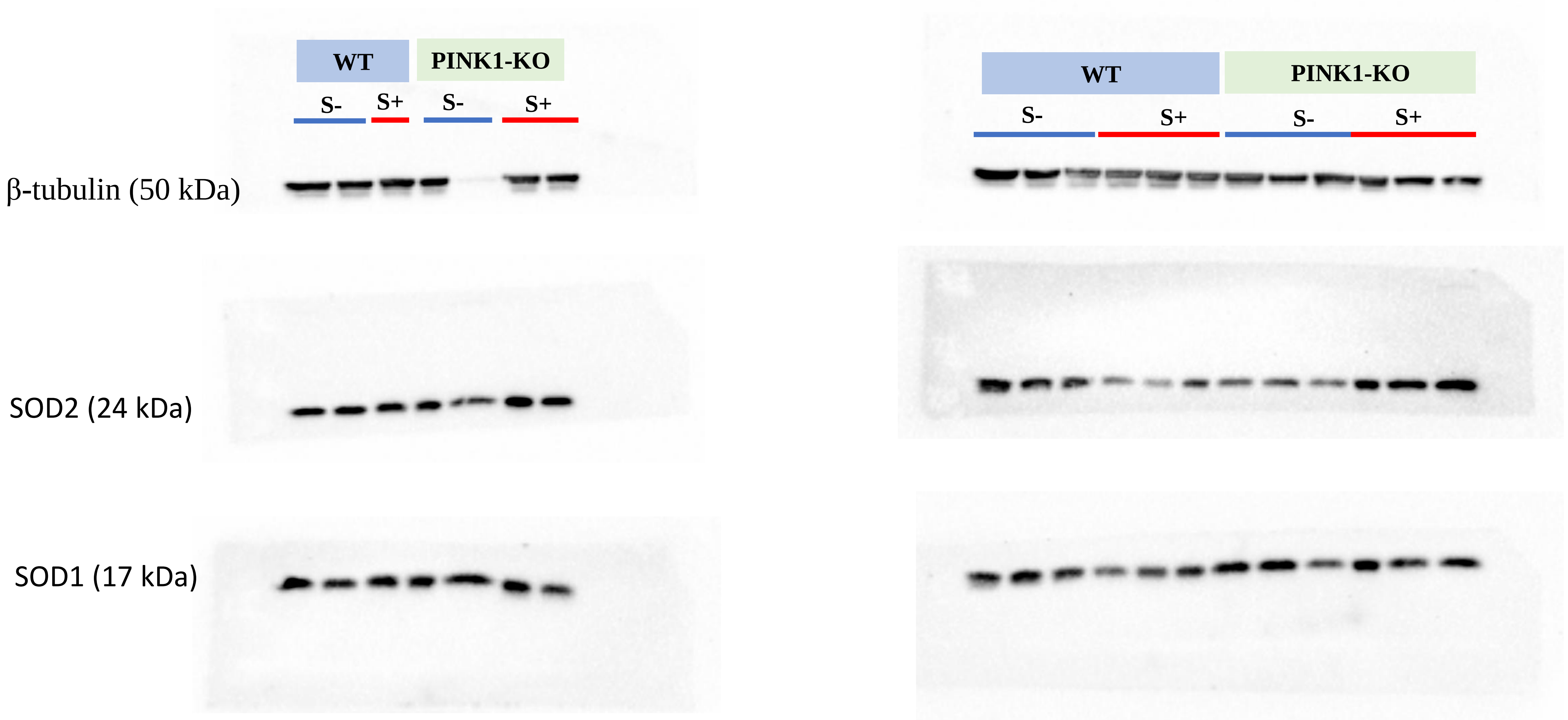

Supplementary Figure S1

FEMALES - Acute effect

Blot 1 _ DJ1 and TOM20

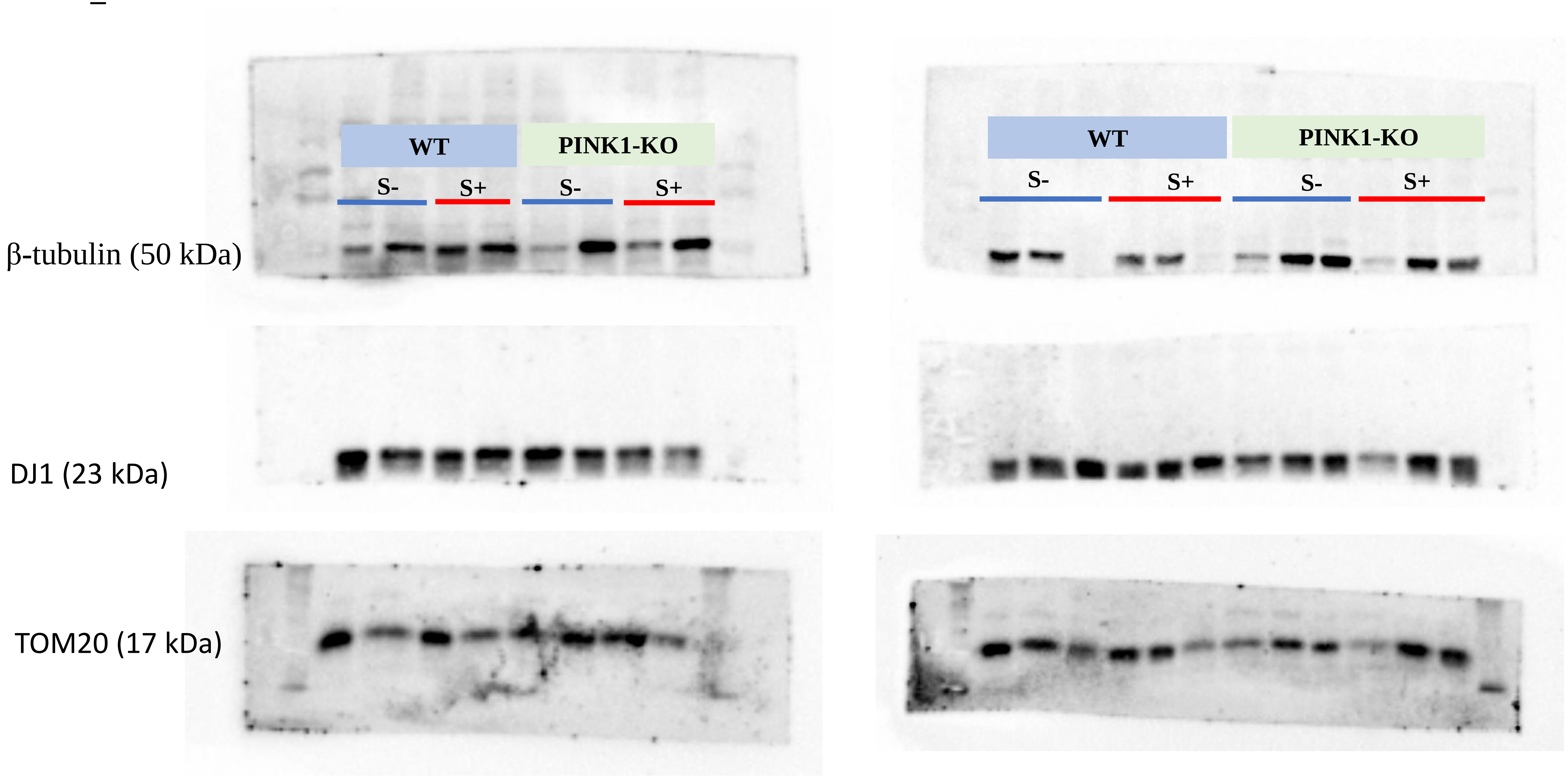

Supplementary Figure S1

FEMALES - Acute effect

Blot 2 for DJ1 and TOM20

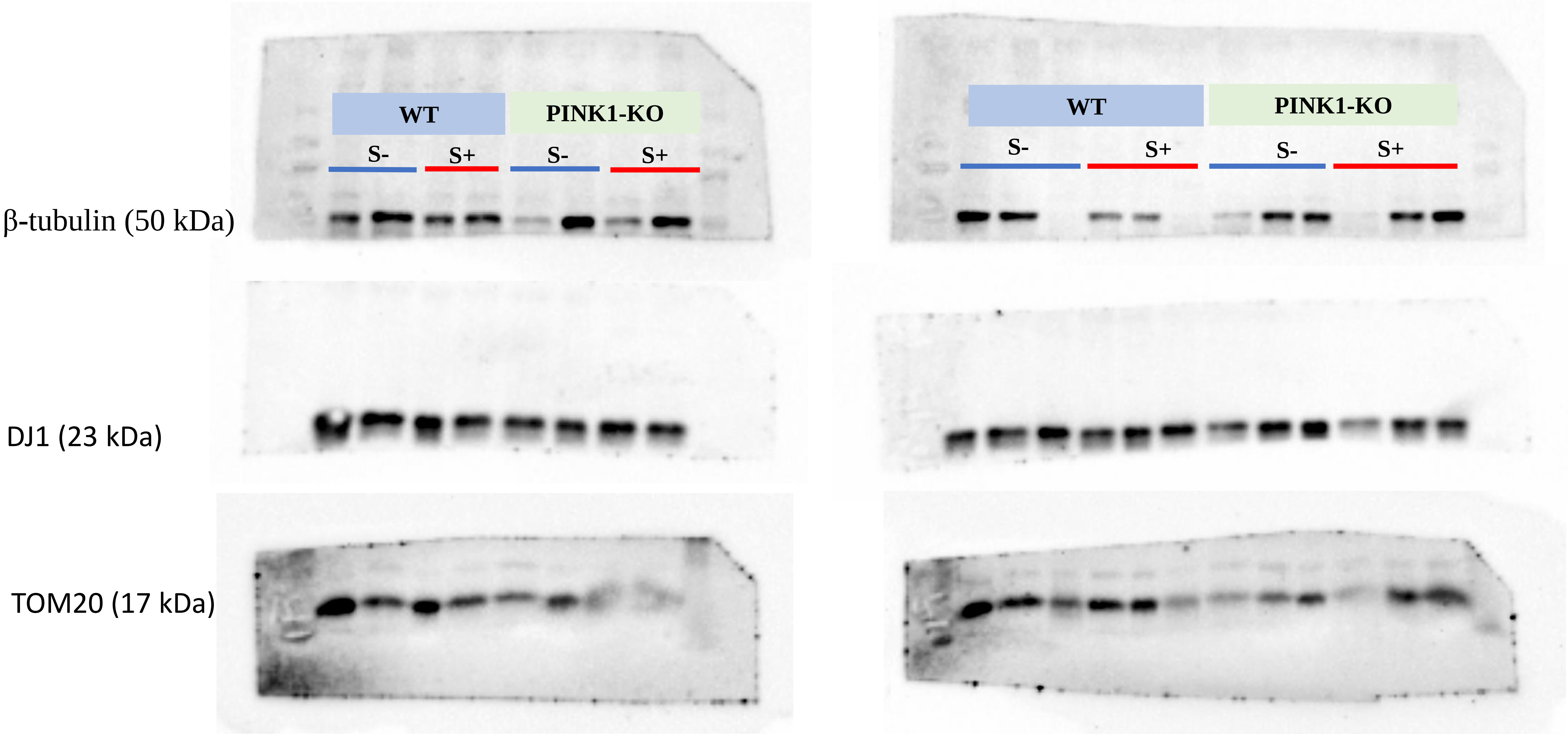

Supplementary Figure S1

FEMALES - Acute effect

Blot 1 for SOD1, SOD2 and BDNF

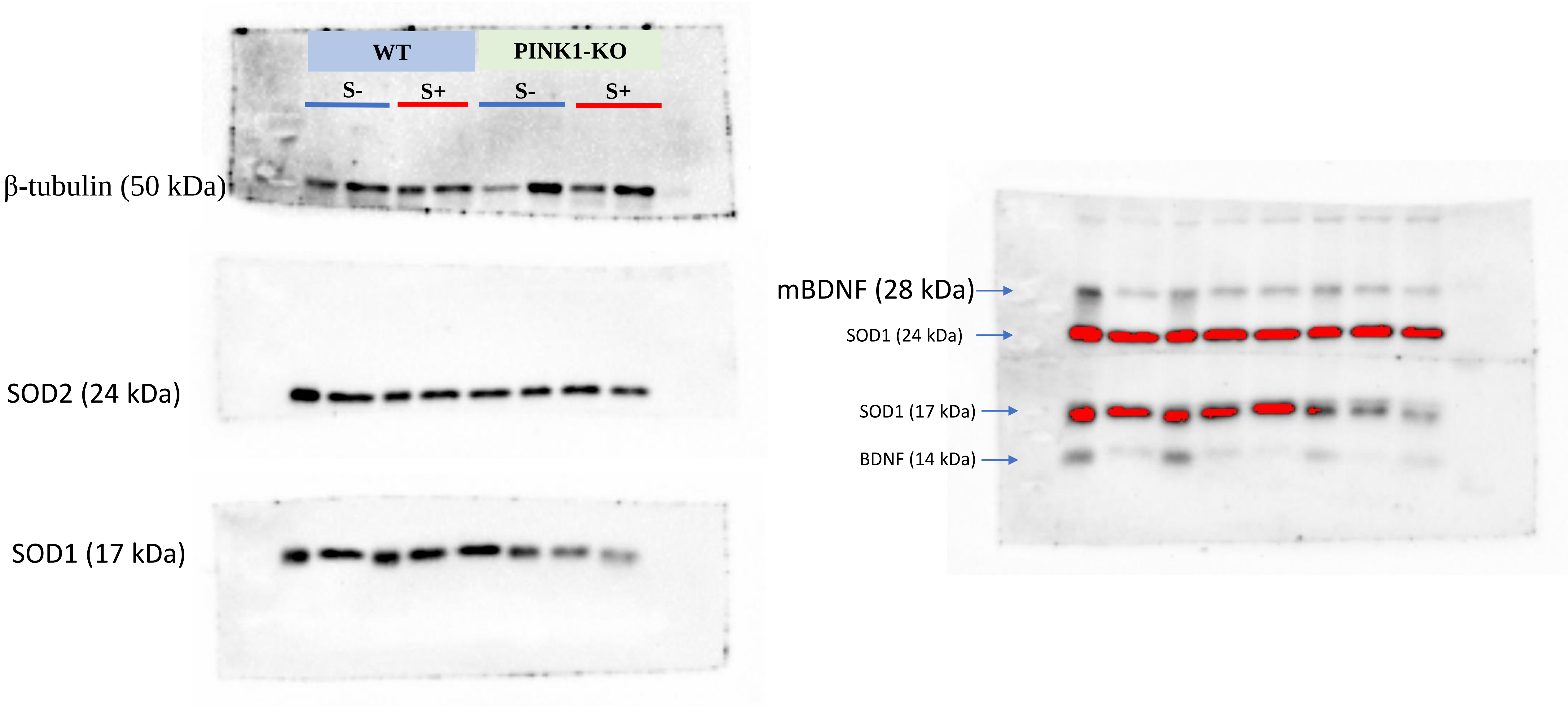

Supplementary Figure S1

FEMALES - Acute effect
Blot 1 for SOD1, SOD2 and BDNF

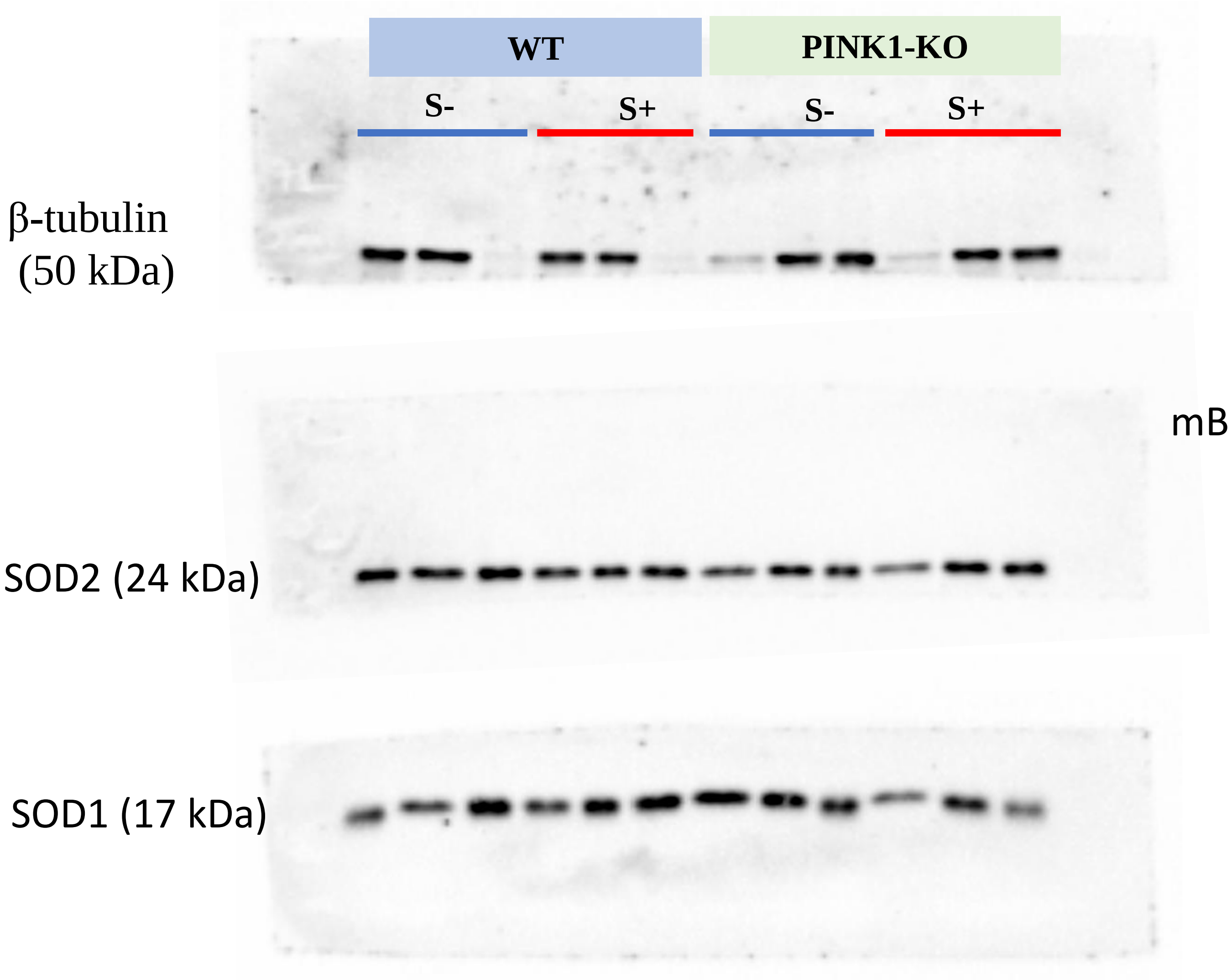

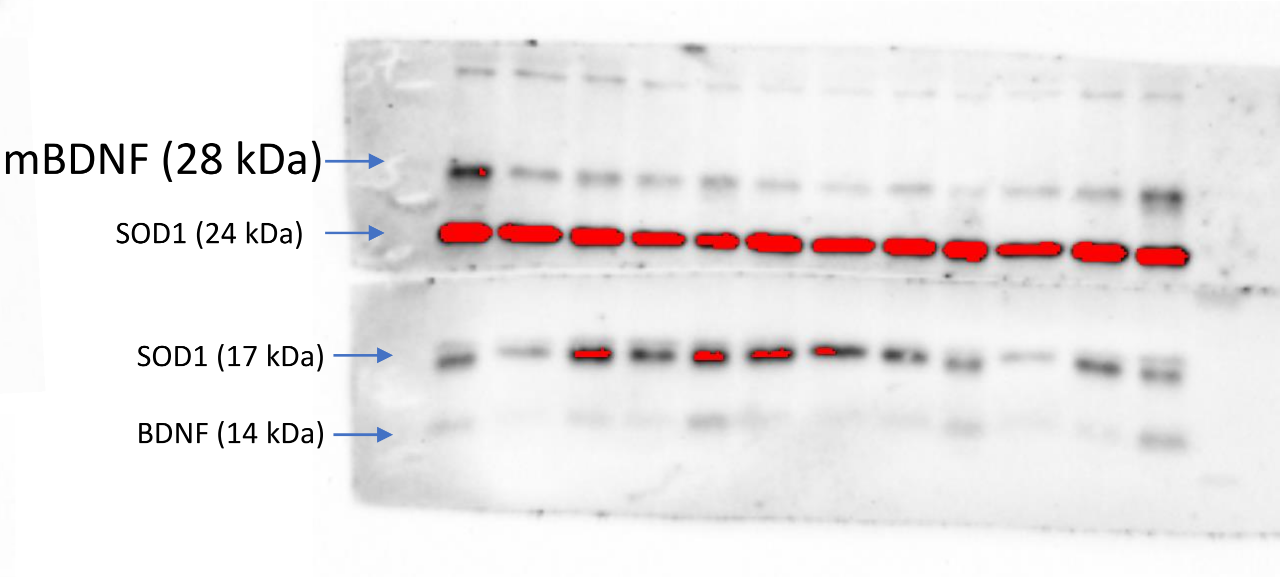

Supplementary Figure S1

FEMALES - Acute effect

Blot 2 for SOD1, SOD2 and BDNF

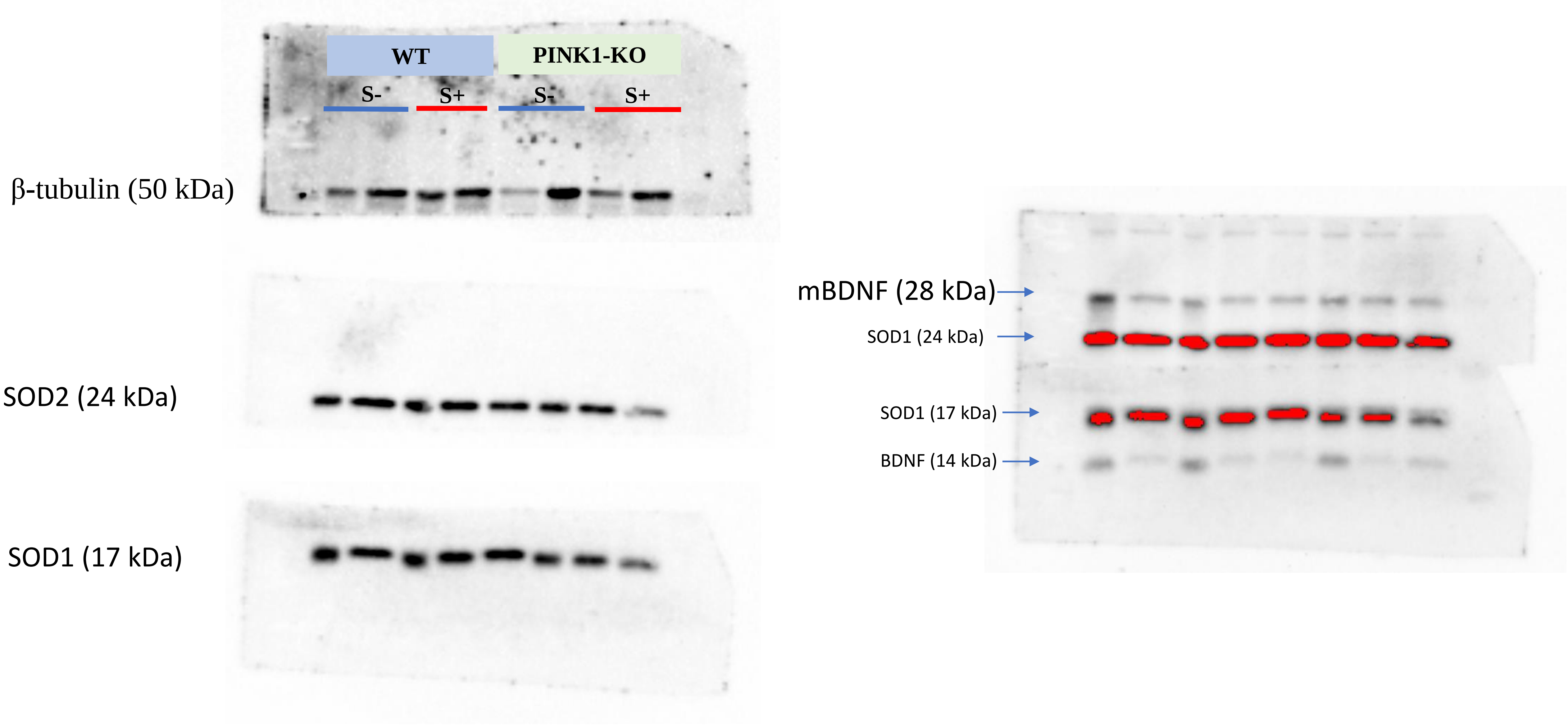

Supplementary Figure S1

FEMALES - Acute effect

Blot 2 for SOD1, SOD2 and BDNF

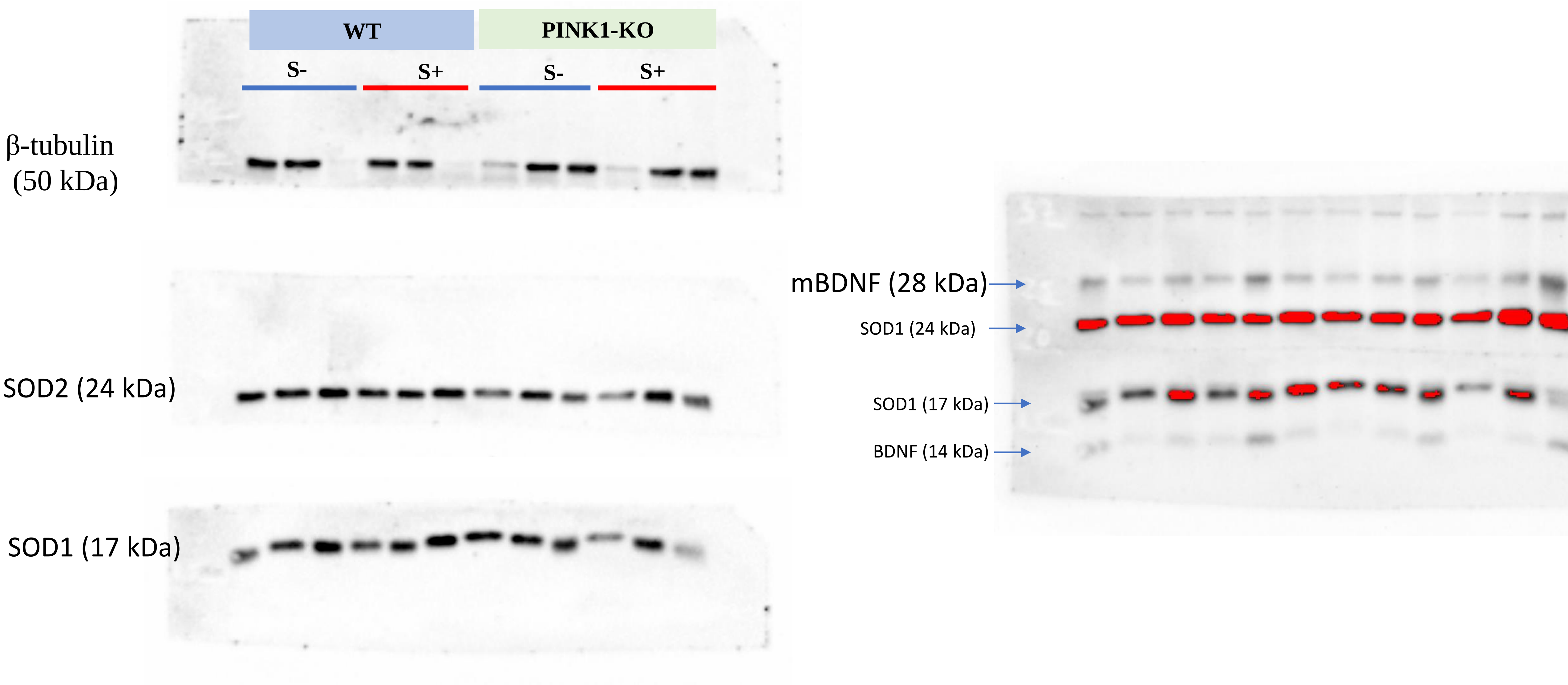

Supplementary Figure S2. OCRs parameters normalized to total protein content of PBMCs from stressed and unstressed PINK1-KO and WT rats. For the assay it was used: 1 µM Oligomycin, 1.5 µM FCCP, 1 µM Antimycin, 100 nM Rotenone. For control groups (S-) n = 14 per group, for stressed groups sacrificed in day 3 the n = 10 per group, and in day 10 the n = 4 per group. Data was normalized to total protein content and expressed as percentage mean ± SEM; *p ≤ 0.05,**p ≤ 0.01, ***p ≤ 0.001, ****p ≤ 0.0001, unpaired t-test.

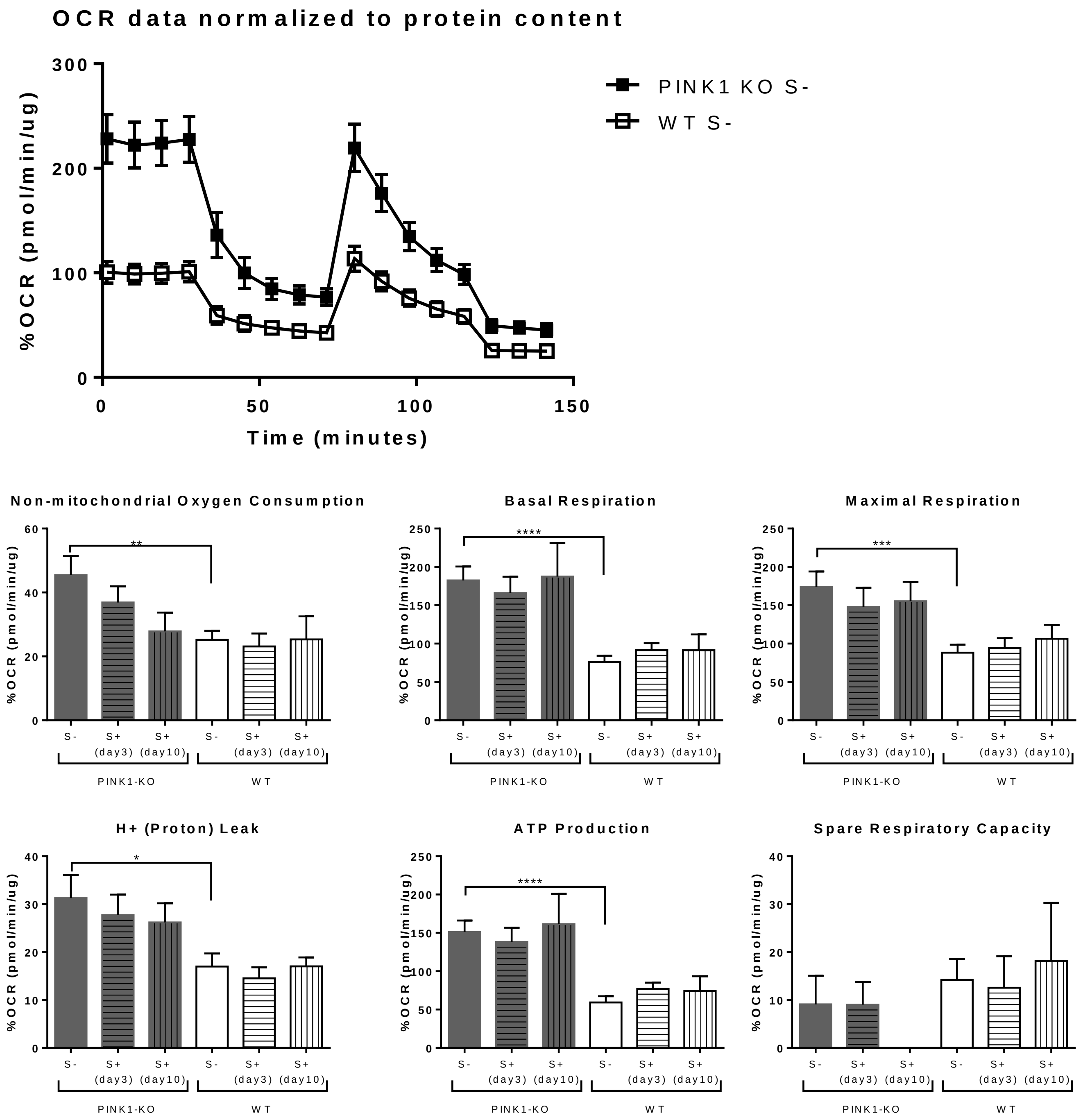

Supplement: Supplementary file 1 — Supplementary data. [file 41598_2020_66745_MOESM1_ESM.pdf]
